# Ipomoeassin-F disrupts multiple aspects of secretory protein biogenesis

**DOI:** 10.1038/s41598-021-91107-4

**Published:** 2021-06-02

**Authors:** Peristera Roboti, Sarah O’Keefe, Kwabena B. Duah, Wei Q. Shi, Stephen High

**Affiliations:** 1 Division of Molecular and Cellular Function, School of Biological Sciences, Faculty of Biology, Medicine and Health, The University of Manchester, Manchester, M13 9PT UK; 2 Department of Chemistry, Ball State University, Muncie, IN 47306 USA

**Keywords:** Protein translocation, Chaperones, Endoplasmic reticulum, Secretion

## Abstract

The Sec61 complex translocates nascent polypeptides into and across the membrane of the endoplasmic reticulum (ER), providing access to the secretory pathway. In this study, we show that Ipomoeassin-F (Ipom-F), a selective inhibitor of protein entry into the ER lumen, blocks the in vitro translocation of certain secretory proteins and ER lumenal folding factors whilst barely affecting others such as albumin. The effects of Ipom-F on protein secretion from HepG2 cells are twofold: reduced ER translocation combined, in some cases, with defective ER lumenal folding. This latter issue is most likely a consequence of Ipom-F preventing the cell from replenishing its ER lumenal chaperones. Ipom-F treatment results in two cellular stress responses: firstly, an upregulation of stress-inducible cytosolic chaperones, Hsp70 and Hsp90; secondly, an atypical unfolded protein response (UPR) linked to the Ipom-F-mediated perturbation of ER function. Hence, although levels of spliced XBP1 and CHOP mRNA and ATF4 protein increase with Ipom-F, the accompanying increase in the levels of ER lumenal BiP and GRP94 seen with tunicamycin are not observed. In short, although Ipom-F reduces the biosynthetic load of newly synthesised secretory proteins entering the ER lumen, its effects on the UPR preclude the cell restoring ER homeostasis.

## Introduction

After synthesis at the endoplasmic reticulum (ER), many soluble proteins including cytokines, hormones and enzymes follow the secretory pathway to the cell surface, where they are released by exocytosis (hereafter called secretory proteins)^1^. Alternatively, having accessed the ER lumen, other soluble proteins are retained there to act as molecular chaperones and folding factors^2^. Soluble proteins destined for the secretory pathway via the ER are typically synthesised as preproteins with cleavable, hydrophobic and highly variable N-terminal signal peptides (SPs)^3,4^. In mammalian cells, most preproteins follow a signal recognition particle (SRP)-dependent co-translational route during their translocation across the ER membrane^5^. Hence, SRP binds to the SP of a newly synthesised preprotein^6^ and targets the ribosome-nascent chain complex to the Sec61 translocon by docking with the SRP receptor^7,8^. The SP of the elongating nascent polypeptide chain then interacts with the central, pore-forming Sec61α subunit of the Sec61 complex^9,10^. Many SPs are sufficiently hydrophobic to induce conformational changes within Sec61α that open its aqueous translocation channel, and thereby provide access to the ER lumen^10,11^. Precursors with less hydrophobic SPs may require assistance from auxiliary components, such as the membrane-embedded TRAP and Sec62/Sec63 complexes, and the ER lumenal chaperone BiP, in order to be efficiently translocated through the Sec61 complex^12–14^. Effective SP engagement and Sec61 channel opening enable efficient polypeptide translocation and provide access to the ER lumenal protein folding machinery. As nascent preproteins are translocated across the ER, SP cleavage occurs at the lumenal side of the membrane, together with modifications including N-glycosylation and disulphide bond formation^15,16^. These events ensure the authentic conformational maturation of fully translocated soluble proteins that may remain ER residents or continue through the secretory pathway.

Cells respond to changes in protein homeostasis (proteostasis) that result from perturbations of ER function by activation of the unfolded protein response (UPR). In mammalian cells, this process may be initiated by activation of one or more of three ER transmembrane stress sensors, IRE1, PERK and ATF6, whose functions are regulated by BiP (reviewed in ref.^17^). UPR activation induces a coordinated transcriptional response, mediated by XBP1s, ATF4 and cleaved ATF6, which alleviates stress by upregulating various ER components including ER lumenal chaperones and other folding factors^18,19^. Concomitantly, PERK-dependent UPR activation transiently attenuates protein synthesis to decrease the load of newly synthesised polypeptides entering the ER^20^. If ER homeostasis cannot be restored, the UPR can switch from cytoprotective to pro-apoptotic signalling, primarily through induction of the transcription factor CHOP, which acts downstream of PERK (reviewed in ref.^21^).

A range of small-molecule inhibitors disrupt SP-dependent opening of the Sec61 translocation channel (reviewed in refs.^22,23^), most likely by binding the Sec61α subunit and preventing SP-mediated conformational changes^24^. Ipomoeassin-F (Ipom-F) is one such potent inhibitor of Sec61-mediated translocation, rendering it highly cytotoxic with IC_50_ values in the low nanomolar range^25^. Furthermore, the cytotoxicity of Ipom-F-derived synthetic analogues correlates with their ability to inhibit protein secretion, suggesting that Ipom-F may cause cell death via its blockade of Sec61-dependent protein translocation^25,26^. Here, we describe the effects of Ipom-F on the biogenesis of soluble secretory proteins using liver-derived HepG2 cells as a model system. The inhibitory effects of Ipom-F on ER translocation vary substantially between different precursor proteins, suggesting that some soluble Sec61 protein clients can bypass its actions at the Sec61 complex. However, continued access to the ER lumen in the presence of the compound does not guarantee protein secretion and our data suggest that Ipom-F treatment also leads to a defect in ER lumenal protein folding. This defect most likely reflects an inability to replenish ER lumenal chaperones that results from two distinct consequences of Ipom-F treatment: firstly, prolonged exposure to Ipom-F results in an atypical ER stress response that fails to effectively upregulate the transcription of mRNAs encoding ER lumenal chaperones; secondly, the Sec61-dependent translocation of several abundant ER chaperones is strongly inhibited by Ipom-F. On the basis of these data, we conclude that Ipom-F directly inhibits protein translocation across the ER membrane and indirectly affects the protein folding capacity of the ER lumen, thereby providing a molecular basis for the atypical UPR that we find it to activate.

## Results

### Ipom-F selectively inhibits the in vitro translocation of soluble Sec61 protein clients

**Figure 1:**
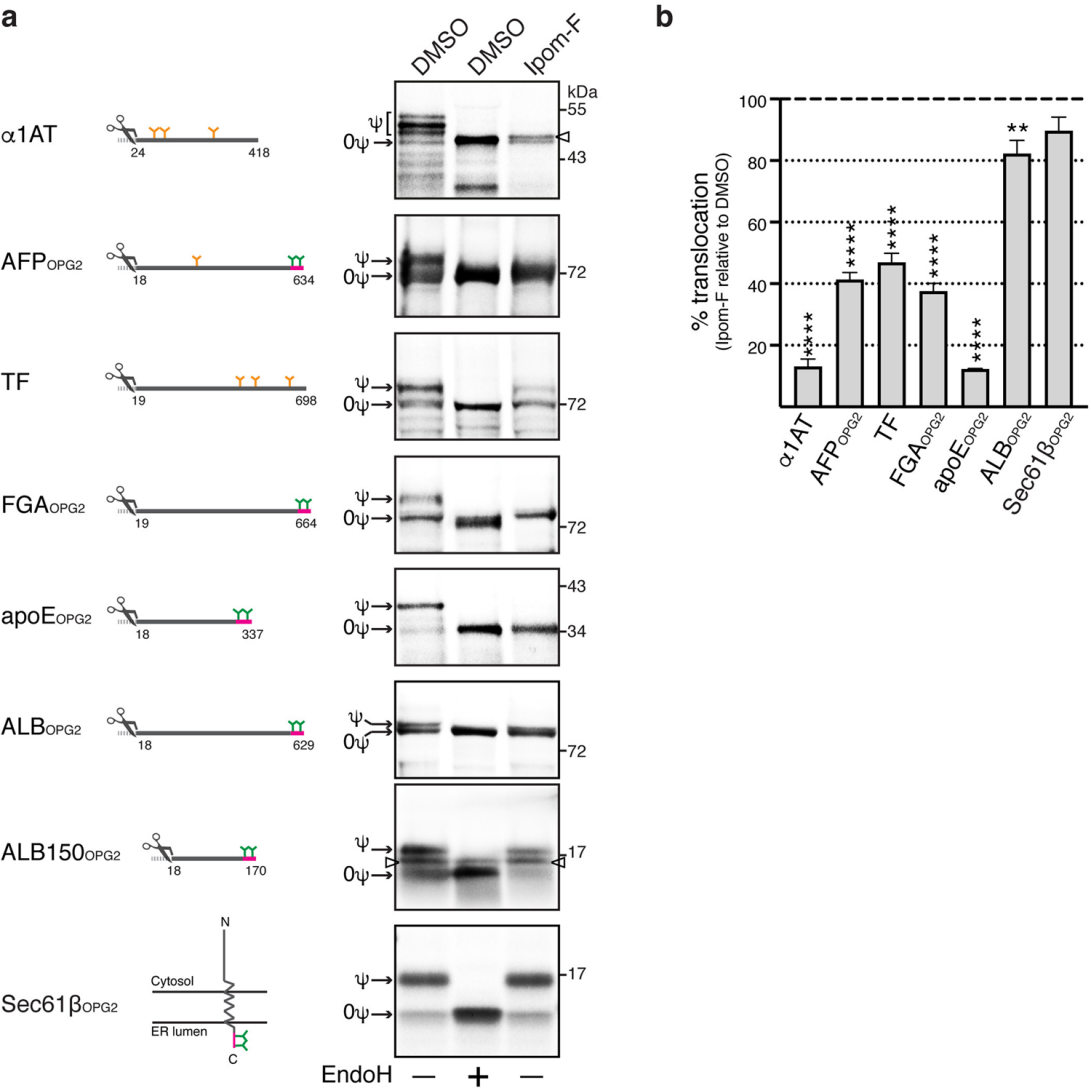
Ipom-F selectively inhibits the in vitro translocation of secretory Sec61 clients. (**a**) α1-antitrypsin (α1AT), serotransferrin (TF) and opsin-tagged variants of α-fetoprotein (AFP_OPG2_), fibrinogen α-chain (FGA_OPG2_), apolipoprotein E (apoE_OPG2_), serum albumin (full-length; ALB_OPG2_, truncated; ALB150_OPG2_) and Sec61β (Sec61β_OPG2_) were translated in rabbit reticulocyte lysate supplemented with [^35^S]Met/Cys, canine rough microsomes and either DMSO or Ipom-F (1 μM). Membrane-associated products were isolated by ultracentrifugation, resolved by SDS-PAGE and analysed directly by phosphorimaging. Control samples were treated with endoglycosidase H (EndoH) to distinguish N-glycosylated (ψ) from non-glycosylated (0ψ) products. The position of a band most likely corresponding to the signal peptide (SP)-uncleaved precursor form of α1AT (see also Supplementary Fig. S1) and ALB150_OPG2_ is indicated with an arrowhead. Representative phosphorimaging exposures are shown (full-length gels presented in Supplementary Fig. S5). Diagrams of constructs used are shown on the left. Numbers represent the corresponding amino acids of the preprotein. The cleavage site of N-terminal signal peptides (scissors symbol) and endogenous N-glycosylation sites (orange Y symbols) are indicated. Where indicated, a 17-amino acid reporter tag (depicted in red) derived from bovine opsin was appended to the C-terminus of proteins to introduce two exogenous N-glycosylation sites (green Y symbols). (**b**) Quantification of the population of in vitro translated substrates from (**a**) that was translocated across the ER membranes following treatment with Ipom-F. Translocation efficiency was determined from the ratio of N-glycosylated to non-glycosylated form for each precursor and expressed relative to the translocation efficiency in DMSO samples (set to 100%). Values are mean ± s.e.m from at least three independent experiments. **P < 0.01; ****P < 0.0001 relative to DMSO (one-way ANOVA).

We reported previously that Ipom-F blocks the translocation of two well-studied secretory proteins, bovine prolactin and yeast pro-α-factor, into canine rough microsomes^25^. To explore the generality of this inhibitory effect, we studied several human secretory proteins that are expressed in HepG2 cells (this study, see also^27^) using the same approach (Fig. 1a). This in vitro assay relies on protein N-glycosylation as a readout for translocation across the ER membrane^25^. Hence, some of these model secretory proteins were engineered to contain a C-terminal opsin tag (OPG2) in order to either enable or enhance the detection of ER lumenal N-glycosylation (Fig. 1a). A quantitative analysis of the translocation of these proteins showed that whilst all of them showed some degree of sensitivity to Ipom-F, there was a wide variation in the defect observed (Fig. 1b). In the case of α1-antitrypsin (α1AT), α-fetoprotein (AFP_OPG2_ denoting presence of an OPG2 tag) and apolipoprotein E (apoE_OPG2_), 1 µM Ipom-F treatment resulted in a substantial loss of translocation (~ 60–90% reduction, Fig. 1b) directly comparable to its published effect on prolactin translocation^25^. In contrast, the inhibition of serum albumin (ALB_OPG2_) translocation seen with Ipom-F was modest and appeared qualitatively similar to the level of Sec61-independent integration of the tail-anchored membrane protein Sec61β_OPG2_ seen in the presence of Ipom-F (Fig. 1a,b; see also^25^). Although full length N-glycosylated ALB_OPG2_ migrates rather closely to its non-glycosylated form, its reduced susceptibility is readily apparent with ALB150_OPG2_, a truncated form of the protein (Fig. 1a, cf. ALB_OPG2_ and ALB150_OPG2_). These in vitro data suggest that the ability of Ipom-F to inhibit the ER translocation of secretory proteins is more variable than our previous, rather limited, studies had suggested^25^.

### Ipom-F inhibits protein secretion in a substrate-selective manner

**Figure 2:**
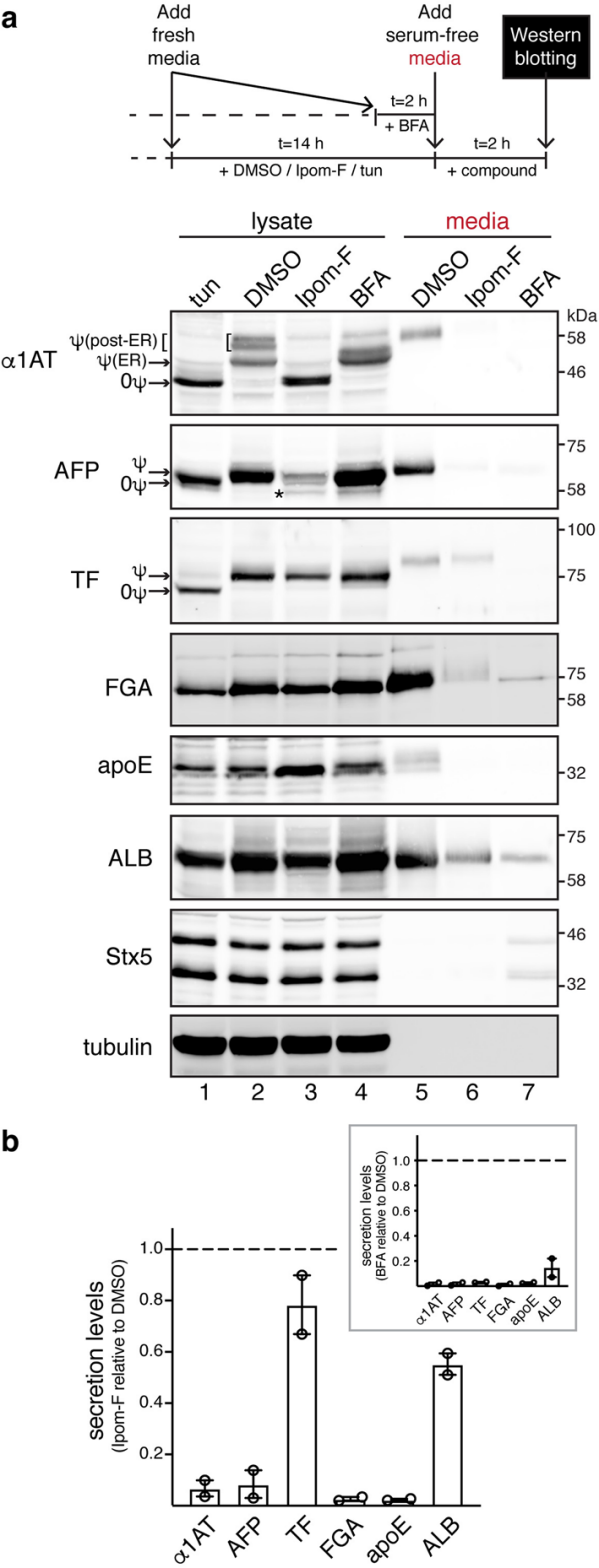
Ipom-F inhibits protein secretion in a substrate-selective manner. (**a**) HepG2 cells were pre-treated either with DMSO, Ipom-F (100 nM) or tunicamycin (tun; 5 μg/ml) for 14 h or with BFA (2.5 μg/ml) for 2 h, followed by treatment in serum-free media for another 2 h in the presence of the compounds as shown in the schematic representation of the assay. Equal amounts of total protein from clarified cell lysates and serum-free media were analysed by immunoblotting for the indicated endogenous secretory proteins or the SNARE syntaxin-5 (Stx5). For the analysis of FGA, 2.5× more of the media sample than lysate sample was loaded to increase FGA signal sensitivity in the media. Tunicamycin treatment effectively prevented N-glycosylation (indicated by ψ) to yield non-glycosylated (0ψ) species. Endogenous α1AT can be observed as three protein bands. The upper bands represent the mature, post-ER forms of α1AT that contain complex N-glycans (reflected by heterogeneity in electrophoretic mobility), while the lower band corresponds to the immature, core-glycosylated form of α1AT located in the ER. The asterisk indicates a truncated form of AFP, previously described in HepG2^70^ and perinatal rat liver^71^ cells, which becomes more prominent after treatment with stress-inducing compounds (see also Fig. 3a). Endogenous Stx5 exists as two isoforms, a 42 kDa-ER and a 35 kDa-Golgi isoform, that result from an alternative initiation of translation^72^. Note that the low levels of Stx5 recovered in media from BFA-treated cells may have been associated with BFA-induced loss of cell integrity. However, no signal from the abundant cytosolic tubulin was detected in these media, suggesting that the release of Stx5 in the media was most likely through unconventional secretion mechanism(s). Representative immunoblots are shown (full-length blots presented in Supplementary Fig. S6). (**b**) Quantification of protein levels in the media harvested from cells treated with Ipom-F or BFA (inset) as shown in (**a**). Secretion levels represent ratios of the protein signal in the media fraction to the corresponding signal in cell lysates (normalised against tubulin). Secretion levels were set to one in DMSO-treated cells. Values are mean ± s.e.m from two independent experiments.

To study the effect of Ipom-F on the same model secretory proteins in a cellular context, we used human, liver-derived HepG2 cells^27,28^. Quantitative immunoblot analysis revealed that the secretion of α1AT, AFP, fibrinogen α-chain (FGA) and apoE was virtually abolished following Ipom-F treatment (Fig. 2a, cf. lanes 5 and 6; Fig. 2b). In contrast, substantial levels of serotransferrin (TF) and ALB were recovered in the media after the inclusion of Ipom-F (Fig. 2a, cf. lanes 5 and 6; Fig. 2b). Since the secretion of all six proteins studied was strongly reduced by the protein trafficking inhibitor brefeldin A (BFA)^29^ (Fig. 2a, cf. lanes 5 and 7; Fig. 2b, inset), we conclude that TF and ALB are unlikely to be released by an alternative exocytic route. The continued, albeit reduced, secretion of TF and ALB (~ 20% and ~ 40% reduction, respectively), most likely results from their ability to bypass the Ipom-F mediated blockade of the Sec61 translocon that we had already established in vitro (see Fig. 1). However, the secretion of AFP and FGA appeared to be more severely impaired by Ipom-F treatment at a cellular level than might be expected from the defects in their ER translocation that were observed in vitro (cf. Figs 1, 2). We therefore conclude that Ipom-F may also influence additional aspects of secretory protein biogenesis and maturation.

By comparing lysates from cells subjected to different treatments, we could distinguish the mature forms of the three secretory N-glycoproteins included in our study from their distinct immature forms (Fig. 2a, cf. lanes 1–4). For α1AT and AFP, Ipom-F treatment resulted in a loss of their respective N-glycosylated species that was accompanied by an increase in the level of faster migrating species that were most likely non-glycosylated (Fig. 2a, α1AT and AFP, cf. lanes 1–4). In contrast, the intracellular pool of N-glycosylated TF appeared unaffected by Ipom-F (Fig. 2a, TF, cf. lanes 1–4), consistent with its continued secretion into the media (Fig. 2a, TF, cf. lanes 5–7, Fig. 2b). Hence, for α1AT, AFP and TF the effects of Ipom-F on their intracellular maturation appear to reflect its consequences for their secretion. Endogenous FGA^30^, apoE and ALB are not modified with N-glycans, making the mature proteins found in the secretory pathway difficult to distinguish from any immature precursors that might result from Ipom-F treatment (Fig. 2a, FGA, apoE and ALB, cf. lanes 1–4). Syntaxin 5 (Stx5) is a tail-anchored protein that utilises a TRC40-mediated, Sec61-independent pathway for its membrane insertion^31^. The level of endogenous Stx5 is unaffected by Ipom-F treatment (Fig. 2a, Stx5, cf. lanes 1–4), consistent with its specificity for Sec61-dependent processes^25^.

### Ipom-F selectively inhibits processing of secretory proteins in the ER lumen

**Figure 3:**
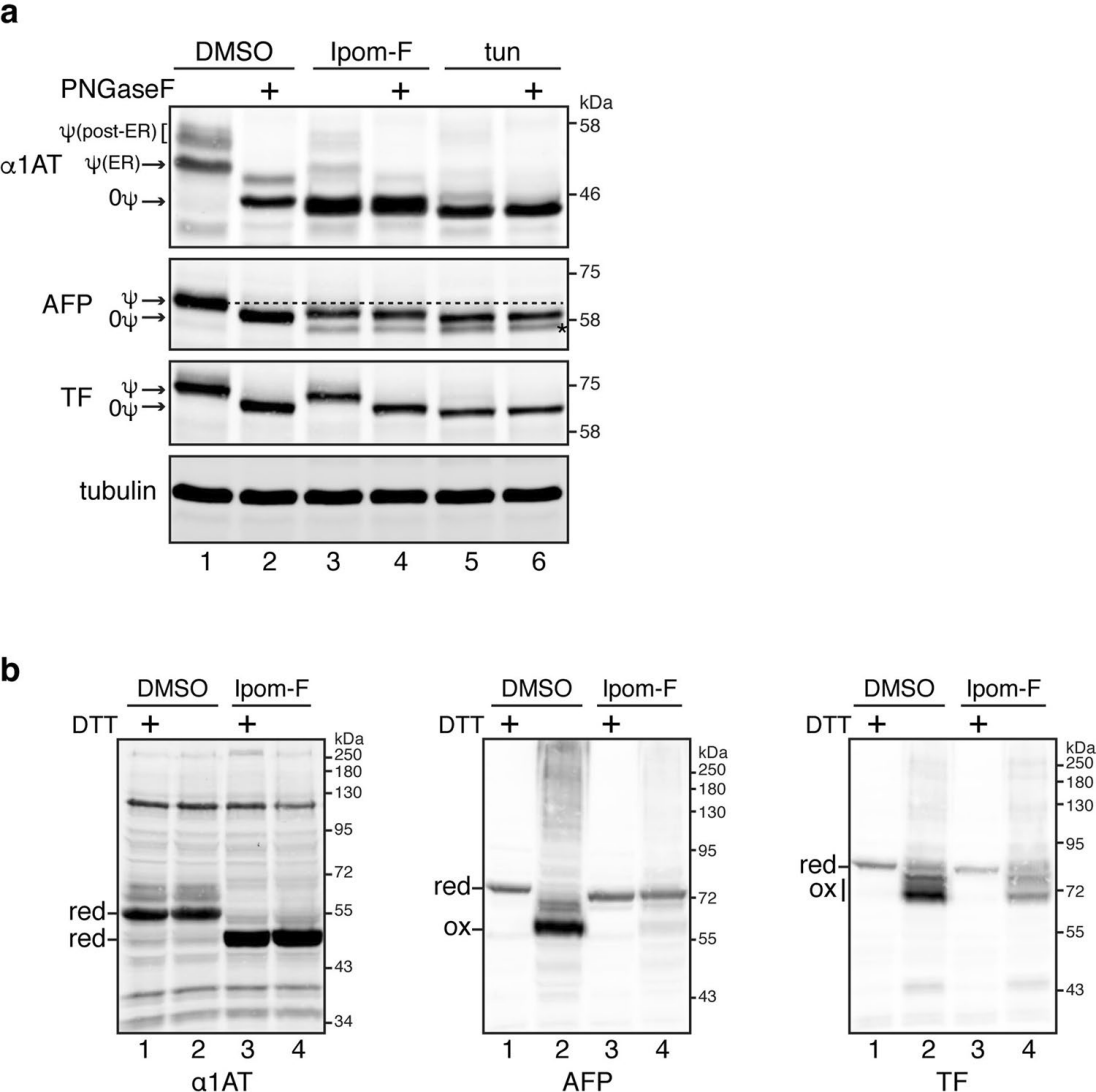
Ipom-F selectively inhibits ER processing of endogenous secretory proteins. HepG2 cells were treated with DMSO, Ipom-F (100 nM) or tunicamycin (tun; 5 μg/ml) for 8 h. Cells were lysed directly with (**a**) reducing sample buffer in the presence or absence of PNGaseF and (**b**) reducing (+ DTT) or non-reducing (-DTT) sample buffer, and analysed by immunoblotting for the indicated model secretory proteins. In (**a**), glycosylated (ψ) and non-glycosylated (0ψ) species are indicated. Post-ER complex-glycosylated and ER resident core-glycosylated α1AT products are shown. Of note, non-glycosylated (0ψ) species of α1AT and AFP, whose translocation was most probably inhibited by Ipom-F, migrate a bit slower than the corresponding species in tunicamycin-treated cells, as predicted by signal sequence cleavage of the latter. The asterisk indicates a truncated form of AFP^70,71^. Representative immunoblots are shown (full-length blots presented in Supplementary Fig. S6). In (**b**), bands corresponding to oxidised (ox) or reduced (red) mature proteins and preproteins are noted.

Having observed an unanticipated level of selectivity for the Ipom-F-dependent blockade of secretory protein translocation (Figs. 1, 2, cf. ref.^25^), we next investigated its effect on protein maturation, focusing on the secretory N-glycoproteins α1AT, AFP and TF. Eight hours treatment with Ipom-F resulted in the accumulation of faster migrating species of both α1AT and AFP, but not TF (Fig. 3a, cf. lanes 1 and 3). These new species were unaffected by treatment with peptide-N-glycosidase F (PNGaseF; Fig. 3a, α1AT and AFP, cf. lanes 3 and 4), confirming they are not N-glycosylated and may therefore correspond to non-translocated precursors that result from Ipom-F treatment. These Ipom-F-dependent products were also compared with tunicamycin-induced, non-glycosylated species that have entered the ER lumen and had their SPs cleaved (Fig. 3a, α1AT and AFP, cf. lanes 3 and 5). Based on subtle differences in the mobility of these Ipom-F-dependent products, we propose that in the case of α1AT and AFP (Fig. 3a, cf. lanes 3–6), they represent full-length precursor proteins that have defaulted to the cytosol and have no access to ER lumenal modifications. In contrast, TF remained sensitive to PNGaseF treatment even in Ipom-F-treated cells (Fig. 3a, cf. lanes 3 and 4). The insensitivity of TF to Ipom-F is underlined by the finding that it is efficiently translocated into the ER lumen even after prolonged treatment with the compound or when used at up to a fivefold higher concentration (Supplementary Fig. S2).

N-glycan-dependent changes in the mobility of endogenous AFP are modest (see Fig. 3a, AFP), and we therefore also analysed the effect of Ipom-F treatment on the ability of newly synthesised secretory proteins to form native disulphide bonds inside the ER lumen^32^. Polypeptides with intact intra-molecular disulphides often migrate faster than their reduced counterparts^33^, as is apparent when comparing products run on SDS-PAGE under reducing (+ DTT) and non-reducing (-DTT) conditions (Fig. 3b). α1AT has no disulphide bonds^34^ and hence its migration pattern on SDS-PAGE was unaltered by DTT (Fig. 3b, α1AT, cf. lanes 1 and 2). In contrast, AFP contains up to fifteen disulphides (Uniprot accession #P02771) and, when lysate from control cells was analysed, its non-reduced form showed increased mobility consistent with successful ER translocation (Fig. 3b, AFP, cf. lanes 1 and 2). Following Ipom-F treatment, this DTT-dependent change in AFP mobility was no longer observed (Fig. 3b, AFP, cf. lanes 3 and 4), indicative of its failure to form native disulphide bonds and confirming the sensitivity of its ER translocation to Ipom-F (cf. Fig. 3a). TF has up to nineteen intra-molecular disulphides (Uniprot accession #P02787), consistent with its increased mobility under non-reducing conditions (Fig. 3b, TF, cf. lanes 1 and 2). However, unlike AFP, these faster migrating TF species were still apparent following Ipom-F treatment (Fig. 3b, TF, cf. lanes 2 and 4). Hence, changes in N-glycosylation and/or disulphide bond formation consistently indicate that TF is less sensitive to the Ipom-F-mediated inhibition of the Sec61 translocon than either α1AT or AFP, and confirm its effect on secretory protein biogenesis is selective.

### Ipom-F-induced secretory protein precursors mislocalise to the cytosol

**Figure 4:**
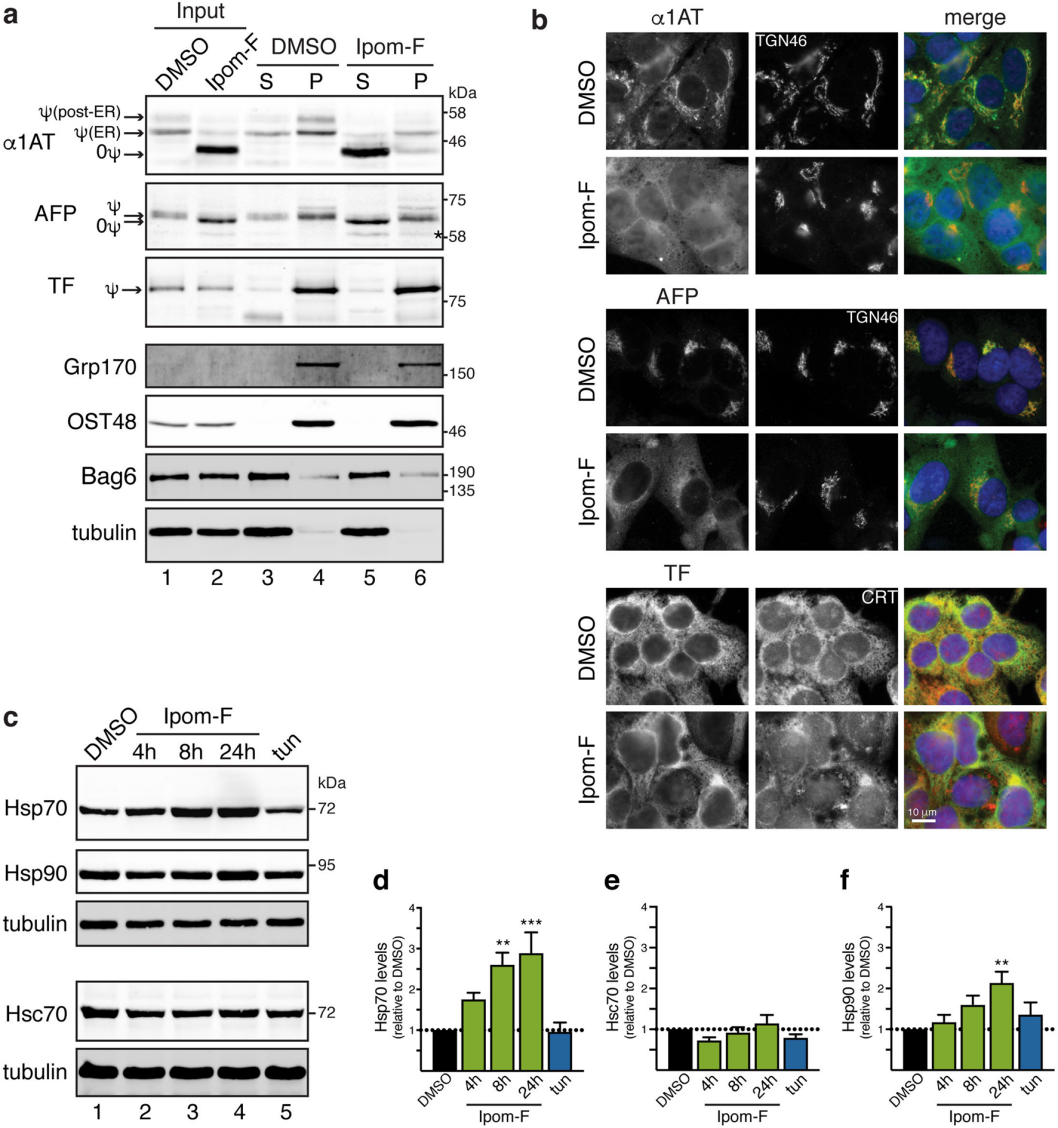
Ipom-F-induced secretory protein precursors mislocalise to the cytosol. (**a**) HepG2 cells, treated with DMSO or Ipom-F (100 nM) for 16 h, were lysed mechanically before separation into supernatant (S) and pellet (P) fractions by ultracentrifugation. Inputs and equivalent amounts of each fraction were analysed by immunoblotting for the indicated proteins. Mature N-glycosylated (ψ) proteins and immature non-glycosylated (0ψ) precursors are indicated. The asterisk indicates a truncated form of AFP^70,71^. Grp170 (ER lumenal marker), OST48 (integral ER membrane marker) and Bag6 (cytosolic marker) remain uniformly in the pellet and supernatant fractions, respectively, and serve as fractionation controls. Full-length immunoblots are presented in Supplementary Fig. S6. (**b**) Representative wide-field fluorescence images of HepG2 cells treated as in (**a**) and immunolabelled against the indicated endogenous Sec61 client (green) and either TGN46 (trans-Golgi network; red) or CRT (ER; red). (**c**) HepG2 cells were treated with DMSO for 24 h, tunicamycin (tun; 5 μg/ml) for 17 h or Ipom-F (100 nM) for the indicated times. Detergent-soluble lysates were blotted for the indicated cytosolic chaperones. Representative immunoblots are shown (full-length blots presented in Supplementary Fig. S6). Please note that the Hsc70 immunoblot shown was also probed with anti-ATF6 antibodies using an Odyssey two colour detection system (LI-COR). Hence, the accompanying tubulin loading control is identical to that shown in Fig. 6e. (**d–f**) Infrared fluorescence-based quantification of chaperone expression as shown in (**c**). Values are mean ± s.e.m; n = 4–6; **P < 0.01; ***P < 0.001 relative to DMSO (one-way ANOVA).

Disruption of protein translocation into the ER can promote the mislocalisation of precursor proteins to the cytosol where they are, in some cases, then degraded^13,35–37^. We therefore examined whether Ipom-F treatment resulted in the mislocalisation of our model secretory N-glycoproteins to the cytosol. Subcellular fractionation of control cells revealed that the vast majority of mature proteins were recovered in the pellet fraction with two ER markers, soluble Grp170 and membrane-embedded OST48 (Fig. 4a, α1AT, AFP and TF, cf. lanes 3 and 4), suggesting that they were in the ER and/or a post-ER, membrane-enclosed compartment of the secretory pathway. The level of mature TF and its localisation to the pellet fraction were not affected by Ipom-F (Fig. 4a, TF, cf. lanes 3–6), consistent with its continued ER translocation and eventual secretion. However, following Ipom-F treatment, non-glycosylated precursor forms of both α1AT and AFP (as seen in Fig. 3a) were readily detected in the supernatant fraction (Fig. 4a, α1AT and AFP, cf. lanes 3–6) together with the bulk of the cytosolic protein quality control factor Bag6. We conclude that Ipom-F prevents the Sec61-mediated translocation of α1AT and AFP, resulting in the mislocalisation of their precursor forms to the cytosol.

To confirm our fractionation studies, we used immunofluorescence microscopy to visualise the localisation of the same endogenous secretory N-glycoprotein cargoes in HepG2 cells. In comparison to control cells, Ipom-F treatment led to a strong reduction of the α1AT and AFP signals that normally localised to ribbon-like structures that were confirmed as the Golgi apparatus by co-staining with TGN46 (Fig. 4b, α1AT and AFP). In most Ipom-F-treated cells, α1AT and AFP staining now showed a more diffuse signal that appeared to be distributed throughout the cytoplasm (Fig. 4b, α1AT and AFP). TF was primarily co-localised with the ER marker calreticulin in control cells, and we found that this pattern was largely maintained following Ipom-F treatment, consistent with its continued translocation into the ER lumen (Fig. 4b, TF). We failed to detect any obvious signs of cytosolic inclusions containing precursor proteins after Ipom-F-treatment, suggesting that non-imported secretory protein precursors may be sequestered and/or degraded before such structures can be formed (cf. ref.^38,39^).

We anticipated that the presence of mislocalised precursors in the cytosol might increase the need for cytosolic chaperones that function to maintain the solubility of such aberrant proteins and route them for degradation^40^. Indeed, Ipom-F treatment caused a time-dependent upregulation of stress-responsive Hsp70s, whereas the levels of their constitutively expressed Hsc70 counterparts remained unchanged (Fig. 4c–e). An upregulation of Hsp90s, which normally function together with Hsp70s to restore proteostasis^41,42^, was also observed (Fig. 4c,f). We speculate that the induction of stress-inducible chaperones in Ipom-F-treated cells reflects the ongoing cytosolic mislocalisation of ER precursors (Supplementary Fig. S3), whose folding is most likely compromised (as seen in Fig. 3b). Consistent with this proposal, we found that the inhibition of protein N-glycosylation following tunicamycin treatment did not significantly induce expression of either Hsp70s or Hsp90s (Fig. 4c,d,f), suggesting that this Ipom-F-dependent upregulation that we observe is not a consequence of the accumulation of misfolded proteins in the ER lumen.

### Ipom-F-mediated Sec61 blockade correlates with cytotoxicity

**Figure 5:**
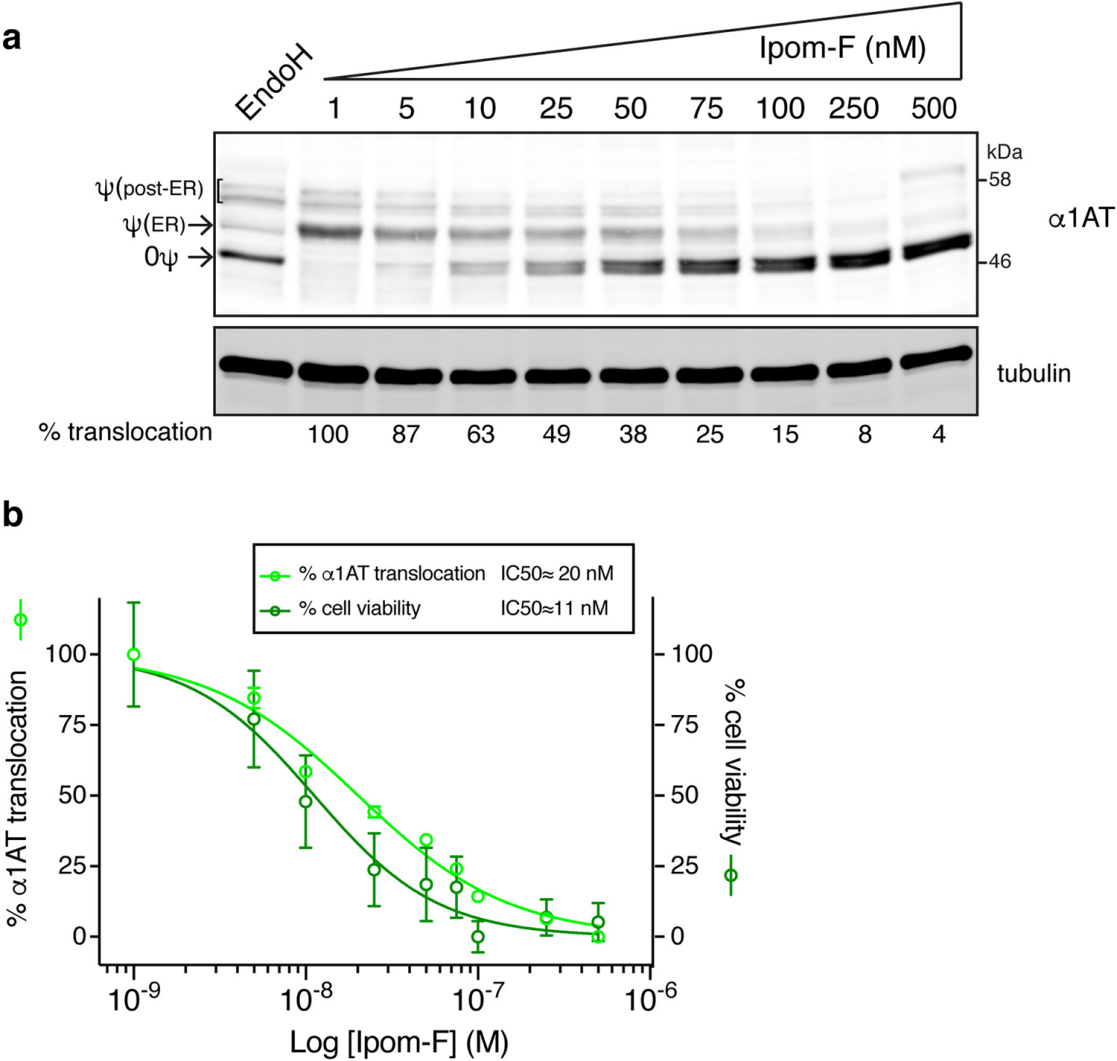
Potent inhibition of α1AT translocation into the ER by Ipom-F correlates with cytotoxicity. (**a**) HepG2 cells treated for 24 h with increasing concentrations of Ipom-F were analysed by immunoblotting for endogenous α1AT. The positions of non-glycosylated (0ψ) and N-glycosylated α1AT (ψ; ER and post-ER species) are indicated. Representative immunoblots shown (full-length blots presented in Supplementary Fig. S6). Infrared fluorescence-based quantification of the efficiency of α1AT translocation into the ER is indicated below the gel. The percentage of α1AT translocation was calculated by dividing the normalised signal intensity of the N-glycosylated species by the sum of N-glycosylated and non-glycosylated species within each lane. (**b**) Dose–response curves for the effect of 24 h Ipom-F treatment on the translocation of endogenous α1AT into the ER (calculated as in (**a**)) and cell viability measured using the MTT assay. Curves are means ± s.e.m of three independent experiments (performed in triplicate for the MTT assay). IC50 (half-maximal effective concentration) values of the dose-responses are shown.

Prolonged treatment of several cell lines with Ipom-F is toxic as a result of its ability to bind and inhibit the Sec61 translocon^25^. We addressed this issue in HepG2 cells by comparing the N-glycosylation of endogenous α1AT with cell viability, upon increasing doses of Ipom-F (Fig. 5). After 24 h of treatment, Ipom-F downregulated cellular ATP levels, as measured by the MTT assay, and inhibited Sec61-mediated protein translocation with comparable potency (Fig. 5b). These data further support the proposal that the blockade of ER translocation is most likely to be the primary molecular basis for the cytotoxic activity of Ipom-F^25,26^.

### Ipom-F activates an unconventional ER stress response

**Figure 6:**
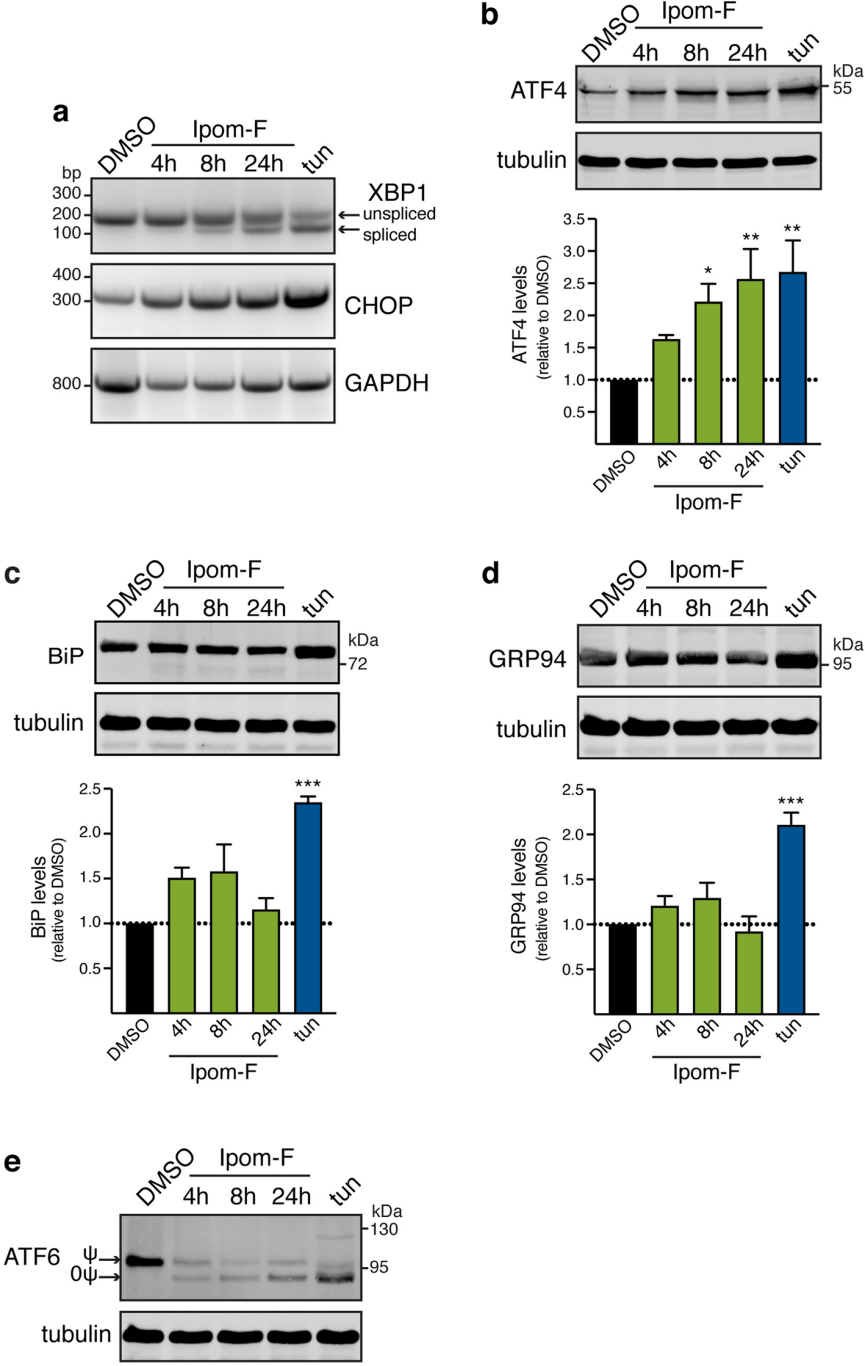
Ipom-F activates an unconventional ER stress response. HepG2 cells were treated with DMSO for 24 h, tunicamycin (tun; 5 μg/ml) for 12 h or Ipom-F (100 nM) for the indicated times. (**a**) Total RNA was isolated from cells, and XBP1 mRNA splicing or levels of CHOP mRNA was determined by reverse transcription-PCR. Unspliced and spliced XBP1 mRNA are indicated. GAPDH served as a cDNA loading control. Full-length agarose gels are presented in Supplementary Fig. S7. (**b–d**) Detergent-soluble lysates were blotted for the indicated proteins. Infrared signals were quantified, normalised against the corresponding tubulin signals and expressed relative to the signal in DMSO-treated cells. Values are mean ± s.e.m; n = 3–4; *P < 0.05; **P < 0.01; ***P < 0.001 relative to DMSO (one-way ANOVA). Representative immunoblots are shown (full-length blots presented in Supplementary Fig. S6). (**e**) Total extracts were blotted for ATF6. Non-glycosylated (0ψ) and N-glycosylated (ψ) ATF6 are indicated. Please note that the immunoblot shown was probed with both rabbit anti-ATF6 and rat anti-Hsc70 antibodies (see Fig. 4c) using two-colour detection with an Odyssey Infrared Imager. Hence, the accompanying tubulin loading control is identical to that shown in Fig. 4c (full-length blots presented in Supplementary Fig. S6).

Small molecule-mediated inhibition of Sec61-dependent protein translocation has previously been linked to ER stress, activation of the UPR and UPR-driven cell death^38,43,44^. Therefore, we next sought to understand how the ER responds to Ipom-F-induced defects in protein translocation. To this end, the effects of increasingly long exposure to Ipom-F were compared to inhibiting protein N-glycosylation with tunicamycin, which leads to a potent activation of the UPR^45,46^. We followed XBP1 mRNA splicing to monitor the IRE1 branch of the UPR^47,48^, detecting splicing after 8 h of Ipom-F treatment that was increased after 24 h exposure (Fig. 6a, XBP1). However, the qualitative extent of splicing appeared weaker for Ipom-F than tunicamycin (Fig. 6a, XBP1). In comparison, a short treatment with the proteasome inhibitor MG-132 (4 h) or longer exposure to the Hsp90 inhibitor 17-AAG (24 h) both failed to induce XBP1 mRNA splicing (Supplementary Fig. S4a), suggesting that the Ipom-F-dependent activation of the UPR is unlikely to be triggered via cytosolic events such as the mislocalisation of non-translocated preproteins to the cytosol (cf. ref.^38^).

For the PERK branch of the UPR, we observed a time-dependent induction of its downstream effector ATF4, such that, after 24 h of Ipom-F treatment, its levels were comparable to cells treated with tunicamycin for 12 h (Fig. 6b). CHOP mRNA levels, which are under ATF4 control, were qualitatively increased over a similar time frame (Fig. 6a, CHOP). The translational upregulation of ATF4 typically occurs under conditions that favour phosphorylation of the translation initiation factor eIF2a^19^. However, under the conditions tested we could not detect any evidence for eIF2a phosphorylation following Ipom-F treatment, despite a clear effect with tunicamycin (Supplementary Fig. S4b).

In order to appreciate the functional consequences of an Ipom-F-driven UPR, HepG2 cells were probed for increased levels of the ER-resident chaperones BiP and GRP94, an established hallmark of ER stress^46^. Whilst tunicamycin-treated cells showed a robust increase in the levels of both proteins, neither was significantly altered by Ipom-F treatment, even after 24 h (Fig. 6c,d). Since the transcription of BiP and GRP94 is regulated through ATF6 signalling^49,50^, we analysed steady-state ATF6 levels following Ipom-F treatment. We observed a rapid qualitative reduction of mature ATF6, concomitant with the appearance of a non-glycosylated form of the protein that co-migrates with the major product seen in tunicamycin-treated cells that are defective for protein N-glycosylation (Fig. 6e). Since Ipom-F inhibits the Sec61-dependent integration of type II membrane proteins like ATF6^25^, we conclude that the ATF6 pathway is likely compromised in Ipom-F-treated cells. This model is further supported by the fact that we find no evidence for any increase in the levels of the mRNAs encoding BiP or GRP94 following Ipom-F treatment, yet exposure to tunicamycin results in a clear qualitative transcriptional upregulation of both (Supplementary Fig. S4c). Taken together, these data argue that Ipom-F treatment produces an atypical ER stress response, which does not involve the upregulation of BiP or GRP94 expression, and is therefore akin to the cellular response previously reported following treatment with mycolactone^44^, another small-molecule inhibitor of the Sec61 complex^22^.

### Ipom-F compromises the translocation of soluble ER residents

**Figure 7:**
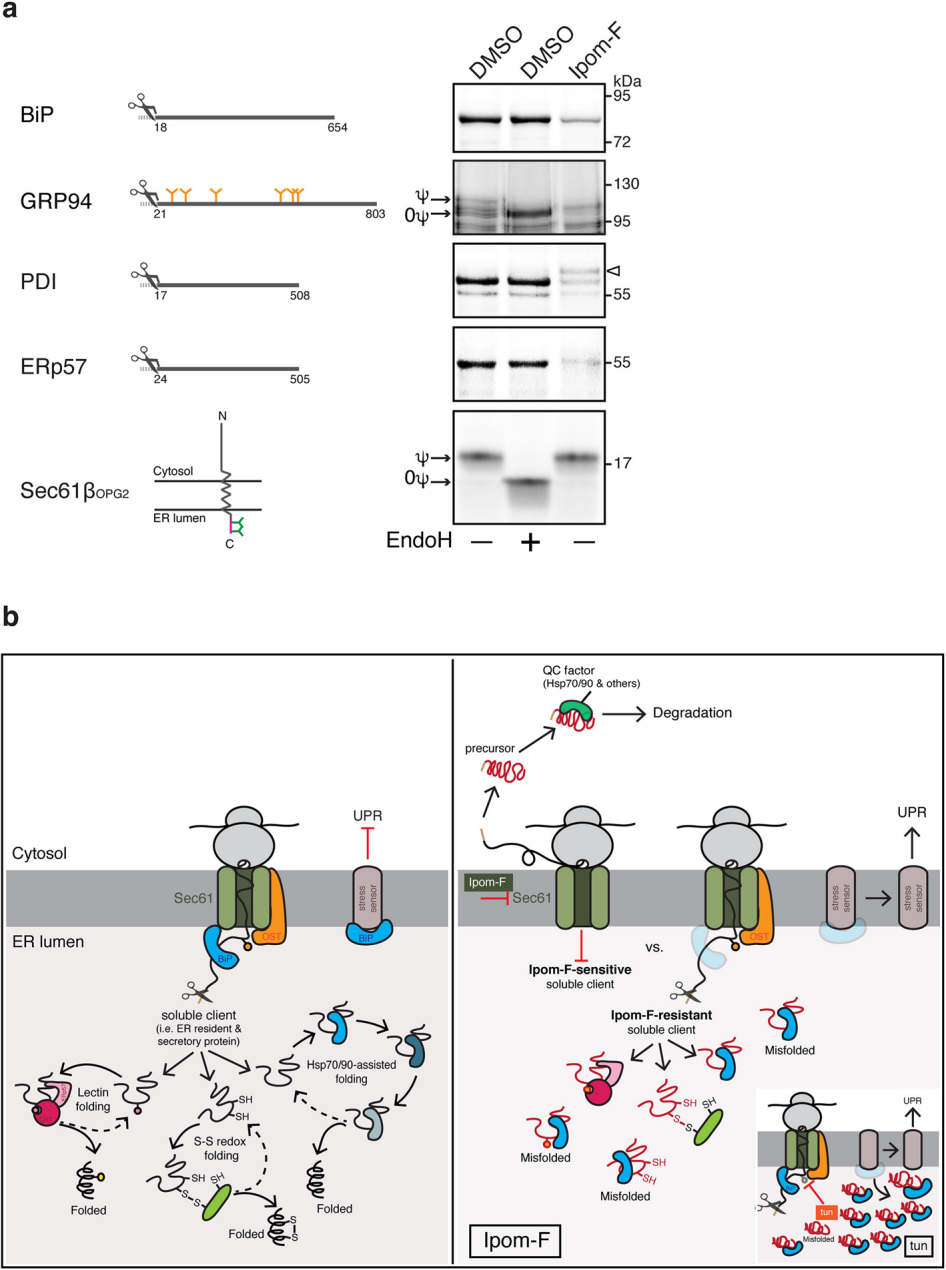
Ipom-F compromises the translocation of soluble ER-resident chaperones and folding factors. (**a**) Model soluble ER-resident folding factors and the tail-anchored protein Sec61β_OPG2_ were translated in rabbit reticulocyte lysate supplemented with [^35^S]Met/Cys, canine rough microsomes and either DMSO or Ipom-F (1 μM), and the membrane-associated products were resolved by SDS-PAGE and analysed directly by phosphorimaging. Control samples were treated with EndoH to distinguish N-glycosylated (ψ) from non-glycosylated (0ψ) products. The position of the SP-uncleaved precursor of PDI is indicated with an arrowhead. Diagrams of preproteins showing the cleavage site of SPs (scissors symbol) and N-glycosylation sites (orange Y sites) are shown on the left. Representative phosphorimaging exposures are shown (full-length gels presented in Supplementary Fig. S5). (**b**) Proposed model of how the disruption of ER import by Ipom-F leads to ER stress. (Left) Under normal conditions, soluble ER clients, i.e. ER lumen residents and secretory proteins, are co-translationally translocated into the ER through the Sec61 translocon, often with the help from auxiliary components such as BiP. Shortly after, they engage ER lumen-resident chaperones and folding factors that promote protein folding and disulphide bond formation. ER stress sensors are maintained in an inactive form through BiP binding^73,74^. Protein N-glycosylation also occurs in the ER lumen during this phase of protein synthesis (not shown for simplicity). (Right) Ipom-F inhibits co-translational import of selected secretory proteins, such as α1AT, leading to the production of non-translocated precursors that mislocalise to the cytosol. These species appear to trigger the induction of cytosolic Hsp70/90 chaperones, which may contribute to a cytosolic quality control mechanism(s). Ipom-F-sensitive substrates also include central ER lumen-resident chaperones (BiP and GRP94) and other folding factors (PDI and ERp57), together with the type II integral membrane proteins ATF6, which acts as an ER stress transducer. These combined defects in ER translocation and ATF6-dependent stress signalling prevent the enhanced level of ER lumenal BiP that is seen after a tunicamycin-induced UPR and perturbs the folding of proteins such as FGA that still access the ER lumen in the presence of Ipom-F. These defects in ER function result in UPR signalling as evidenced by XBP1 mRNA splicing and increased levels of CHOP mRNA and ATF4 protein. (Right-inset) Tunicamycin inhibits protein N-glycosylation, which leads to the accumulation of incorrectly folded proteins in the ER. Increased demand for protein folding and degradation induces ER stress and activates UPR signalling. Dissociation of BiP from the ER stress sensors has been proposed to activate the UPR^73,74^.

Our findings suggest that a potent inhibition of the Sec61 translocon caused by Ipom-F and the resultant depletion of cellular ATF6 levels contribute to the strong perturbation of normal ER function. Given that the ER translocation of several secretory proteins is sensitive to Ipom-F (see Figs. 1, 2, 3, 4), we hypothesised that defects in the translocation of soluble, ER-resident chaperones might also contribute to a loss of ER function. We therefore used our in vitro assay to directly assess the effect of Ipom-F on the translocation of representative ER lumenal chaperones and folding factors. The translocation of both BiP and GRP94 into ER-derived microsomes was strongly reduced by Ipom-F (Fig. 7a). Likewise, the in vitro translocation of two major oxidoreductases, PDI and ERp57, was greatly diminished. In contrast, the Sec61-independent integration of the tail-anchored protein Sec61β_OPG2_ was again unaffected (Fig. 7a, see also Fig. 1a). Thus, in addition to inhibiting the UPR-mediated upregulation of molecular chaperone expression, most likely via its perturbation of ATF6 (see Fig. 6c,d,e), Ipom-F also prevents the translocation of newly synthesised chaperones and folding factors into the ER lumen. Taken together our data show that Ipom-F impacts on chaperone-mediated protein folding and quality control in both the ER lumen and the cytosol, the combined effects of which result in a cytotoxic imbalance of cellular proteostasis.

## Discussion

In this study, we characterise the effect of the Sec61α inhibitor Ipom-F on the release of endogenous secretory proteins from HepG2 cells^51,52^. We find that Ipom-F differentially impairs the ER translocation of these Sec61 clients, resulting in the activation of an atypical ER stress response. In the simplest cases, namely α1AT, AFP and apoE, we find that Ipom-F strongly inhibits their Sec61-dependent translocation into the ER (by ~ 60% or more, see Fig. 1) and this is mirrored by a strong inhibition of their secretion from HepG2 cells (~ 90% or more, see Fig. 2). Likewise, the ER translocation of TF, and even more so ALB, are refractive to Ipom-F treatment (Fig. 1) and substantial amounts of both proteins continue to be secreted by HepG2 cells treated with Ipom-F, but not BFA (Fig. 2). Amongst the pool of secretory proteins studied, the behaviour of FGA is particularly revealing. Hence, although its resistance to the Ipom-F-mediated inhibition of Sec61 is comparable to TF (~ 60% reduction, see Fig. 1), at a cellular level Ipom-F causes an almost complete loss of FGA secretion (~ 95% reduction, see Fig. 2). We therefore conclude that Ipom-F can also affect the biogenesis of some secretory proteins at a point after they have been translocated into the ER lumen, most likely by impacting on their chaperone-mediated folding (see below).

Whilst many Sec61 inhibitors including mycolactone^44,53^, apratoxin A^54^ and coibamide A^55^ are reported to have wide-ranging effects on ER translocation, others like cotransin (CT8) are more substrate-selective^56^. Our current study shows that Ipom-F also displays a previously unappreciated^25^ degree of substrate specificity with regards to its effects on both ER translocation and protein secretion. Ipom-F binds strongly, yet reversibly, to Sec61α^25^, most likely blocking the protein translocation pathway normally initiated by SP binding (cf. ref.^24^). We speculate that in the case of Ipom-F-resistant proteins, their SPs may be able to compete with Ipom-F for Sec61 binding, and thereby displace the inhibitor to allow ER membrane translocation. In cellulo studies of α1AT and AFP confirm a loss of N-glycosylation (Fig. 3) concomitant with the mislocalisation of their immature forms to the cytosol (Fig. 4a,b). In contrast, TF continues to access the ER lumen in the presence of Ipom-F, enabling it to be correctly N-glycosylated and disulphide bonded (Figs. 3, 4) consistent with its continued secretion (Fig. 2). Although mislocalised proteins can be aggregation-prone^39,57,58^, we find no evidence of cytosolic inclusions suggesting that the secretory protein precursors that are mislocalised via Ipom-F can be removed or sequestered via cytosolic quality control pathways^35–38,59–61^. This possibility is also consistent with the significant upregulation of cytosolic Hsp70s and Hsp90s that we observed in Ipom-F-treated cells (Fig. 4c–f), reminiscent of effects previously described following Sec61α depletion^12^ or inhibition with mycolactone^62,63^. We propose that the upregulation of these chaperones most likely represents a cellular defence mechanism that contributes to the recognition and removal of such mislocalised secretory proteins.

Our parallel examination of the effects of Ipom-F on the membrane translocation of endogenous α1AT and the viability of HepG2 cells (Fig. 5), supports the suggestion that the cytotoxic properties of Ipom-F are directly related to its inhibition of Sec61^25^. Our studies also addressed the important question as to whether Ipom-F induces an ER stress response. We find that Ipom-F treatment causes XBP1 mRNA splicing, indicative of IRE1 activation (Fig. 6a), and upregulates the expression of both ATF4 and pro-apoptotic CHOP (Fig. 6a,b). However, in contrast to a typical ER stress response, as seen after tunicamycin treatment^46^, we see no significant increase in the levels of the ER lumenal chaperones BiP and GRP94 with Ipom-F (Fig. 6c,d). Although pre-existing BiP and GRP94 are comparatively long-lived^64^, our data reveal that after Ipom-F treatment, HepG2 cells face two distinct impediments to a UPR-mediated increase in the levels of these chaperones. Firstly, the rapid onset of ATF6 depletion following Ipom-F treatment (by 4 h, see Fig. 6e) is mirrored by a selective failure to upregulate BiP and GRP94 mRNA production (Supplementary Fig. S4c). Secondly, if any newly synthesised BiP and GRP94 are produced, Ipom-F inhibits their SP-mediated, Sec61-dependent translocation into the ER lumen (Fig. 7a). Given that BiP and GRP94 support the folding of newly translocated polypeptides as they enter the ER lumen^65^, whilst BiP also regulates opening of the Sec61 translocon^13,66^, the resulting perturbations of ER lumenal chaperones are likely to further compromise ER function. In the case of the secretory protein FGA, we speculate that it is defects in its chaperone-mediated protein folding and/or ER exit that most likely prevent its secretion, despite the partial resistance of FGA to the Ipom-F-mediated inhibition of ER translocation. In short, our findings establish that Ipom-F alters the pool of secretory proteins that can access the ER lumen whilst also limiting the availability of the ER chaperones that typically promote protein folding and maturation inside the ER lumen (Fig. 7a). However, our data also suggest that any effects on ER lumenal oxidoreductases, such as PDI and ERp57 (Fig. 7a), may be substrate specific, since TF appears to remain correctly disulphide bonded after Ipom-F treatment (Fig. 3b).

In terms of the ER stress that we observe, our current working model is that Ipom-F induces this response by inhibiting the biogenesis of ER chaperones and/or components of the UPR (Fig. 7b). Since some secretory proteins, as evidenced by ALB and TF, continue to access the ER lumen even in the presence of Ipom-F, the resulting perturbation of ER function may eventually result in the misfolding and ER accumulation of at least some secretory protein cargoes, which could also contribute to activation of the UPR. The ability of Ipom-F to recapitulate many features of a tunicamycin-induced UPR (Fig. 6) supports such a model (Fig. 7b, right), although it seems likely that the multifaceted disruption of ER lumenal BiP levels that we report here may contribute to a chronic activation of the UPR. Likewise, the depletion of ATF6 (Fig. 6e) that most likely results from the effects of Ipom-F on its membrane insertion^25^ would further compromise the ability of the cell to reprogram the expression of ER components and correct defects in protein folding and quality control^50,67^. Hence, although Ipom-F reduces the load of newly synthesised secretory proteins that enter the ER lumen, it also debilitates the ability of the cellular UPR to restore ER homeostasis, and thereby most likely promotes apoptotic cell death.

## Methods

### Reagents

Ipom-F was synthesised as previously described^25^. All standard laboratory reagents were from Sigma-Aldrich, unless otherwise stated.

### Cell culture and treatment

HepG2 cells (passage 2) were purchased from ATCC and maintained in DMEM supplemented with 10% (v/v) FBS at 37 °C in a 5% CO_2_ humidified atmosphere. Unless otherwise noted, Ipom-F was used at 100 nM final and the DMSO vehicle control was diluted to the same extent (i.e. final concentration of 0.1% v/v). Cells were treated with 5 μg/ml tunicamycin for up to 17 h or 2.5 μg/ml BFA. Other compounds used were MG-132 (10 μM final) and 17-AAG (Selleckchem; 0.5 μM final). Cells were generally seeded at a density of 0.5 million per well in a six-well plate and grown for 3 days before further processing.

### Analysis of protein secretion

Semi-confluent cells seeded in 6 cm dishes were incubated with DMSO, Ipom-F or tunicamycin for 14 h, whereas pre-treatment with BFA were performed for 2 h. After washing twice with PBS, cells were incubated in serum‐free DMEM for 2 h in the continued presence of the compounds. The medium was then collected and centrifuged at 380×*g* for 5 min at 4 °C to remove cellular debris before overnight precipitation of secreted proteins with 13% (v/v) TCA at − 20 °C. TCA precipitates were washed in ice-cold acetone and dissolved in 2 × SDS-PAGE sample buffer. Cells were solubilised with Triton X‐100 lysis buffer (20 mM Tris–HCl pH 7.5, 150 mM NaCl, 1% (v/v) TX-100, 1 mM EDTA, 1 mM EGTA, 2.5 mM Na_4_P_2_O_7,_ 1 mM Na_3_VO_4_ and 2 mM β-glycerophosphate) supplemented with 1 mM PMSF and a protease inhibitor cocktail. Clarified lysates and media corresponding to ~ 100 μg total intracellular protein were analysed by immunoblotting.

### Protein extraction and western blot

Cells were solubilised in RIPA buffer (50 mM Tris–HCl pH 7.5, 150 mM NaCl, 1% (v/v) NP-40, 1 mM EDTA, 1 mM EGTA, 0.1% (v/v) SDS and 0.5% (w/v) sodium deoxycholate) supplemented with 1 mM Na_3_VO_4_, 2 mM β-glycerophosphate, 25 mM NaF, 1 mM PMSF and a protease inhibitor cocktail by shaking at 4 °C for 1 h. Detergent-insoluble material was cleared by centrifugation at 17,000×*g* for 30 min at 4 °C. For ATF6 detection, cells were directly solubilised in SDS-PAGE sample buffer to ensure that the nuclear pools of ATF6 were efficiently solubilised. Cell lysates were digested with 500 units of PNGaseF (New England Biolabs) in 1× SDS sample buffer for 20 h at 37 °C.

For analysis of disulphide-linked species at steady-state, cells were incubated twice with PBS supplemented with 20 mM NEM for 10 min on ice and then lysed directly with non-reducing SDS-PAGE sample buffer supplemented with 20 mM NEM in order to alkylate free sulphydryl groups, thereby preventing post-lysis, artificial disulphide bond formation. After denaturation at 95 °C for 10 min, half of the lysate was reduced with 20 mM DTT and heating at 95 °C for a further 5 min. Subsequently, samples were allowed to cool to room temperature and NEM was added to 50 mM final into both sets of samples to enable loading of reduced and non-reduced samples next to each other^33^.

Proteins were separated by SDS-PAGE and transferred to PVDF membranes (Immobilon-FL; Merck) for analysis by infrared immunoblotting. Primary antibodies were diluted (as described in the Supplementary Table S1) in a casein-based blocking buffer supplemented with 0.1% (v/v) Tween-20. IRDye 680LT/800CW-conjugated secondary antibodies raised in donkey were purchased from LI-COR and used at 1/5000 in blocking buffer supplemented with 0.1% (v/v) Tween-20 and 0.01% (v/v) SDS. The fluorescent bands were visualised on the Odyssey Infrared Imager (LI-COR Biosciences) and quantified using the Image Studio software provided by the manufacturer.

### Subcellular fractionation

Cell pellet from two confluent 15 cm dishes was washed twice with ice-cold buffer A (10 mM HEPES–KOH pH 7.4, 3 mM Mg(OAc)_2_, 5 mM EGTA and 250 mM sucrose) and subsequently resuspended in 1 ml of buffer A supplemented with a protease inhibitor cocktail and 1 mM PMSF. The cell suspension was kept on ice for 30 min and then homogenised by 20–25 passes through a cell homogenizer (Isobiotec, Germany) with a tungsten carbide ball (clearance of 10 μm). A post-nuclear supernatant fraction (input) was obtained by two rounds of centrifugation at 85×*g* for 10 min at 4 °C. Next, the post-nuclear supernatant was loaded on top of an ice-cold sucrose cushion (25% w/v sucrose in buffer A) and centrifuged at 106,000×*g* for 30 min in a TLS-55 rotor (Beckman Coulter) at 4 °C. The supernatant fraction was removed carefully and the pellet was washed with Buffer A, then centrifuged again at 106,000×*g* for 20 min at 4 °C and solubilised directly into SDS-PAGE sample buffer.

### Immunofluorescence

Cells growing on coverslips were fixed with 4% (v/v) PFA in PBS for 20 min at room temperature, and then permeabilised with 0.1% (v/v) Triton X-100 for 10 min. Coverslips were washed with PBS, and primary antibody (listed in the Supplementary Table S1) and secondary antibody incubations were performed in PBS (1/1000 dilution) at room temperature for 1 h each. Alexa Fluor 488/594-conjugated donkey α-rabbit, α-sheep and α-chicken antibodies were purchased from Invitrogen. The DNA dye DAPI was included during the incubation with the secondary antibodies. Coverslips were mounted in ProLong Gold (Invitrogen), and analysed using an Olympus BX60 upright microscope equipped with a MicroMax cooled, slow-scan CCD camera (Roper Scientific) driven by Metaview software (University Imaging Corporation). Images were processed using Adobe Photoshop CS5.

### RT-PCR analysis of XBP1 mRNA splicing and CHOP mRNA induction

Total RNA was extracted from cells using Isolate II RNA kit (Meridian Bioscience) according to the manufacturer’s instructions. DNase-treated RNA (1 μg) was reverse-transcribed into single-stranded cDNA using the AMV-RT system (New England Biolabs), and cDNA was used as template for PCR amplification of XBP1, CHOP, BiP, GRP94 or GAPDH mRNA. Primers used for RT-PCR analysis included: XBP1, 5′-ACAGCGCTTGGGGATGGATG-3′ and 5′-TGACTGGGTCCAAGTTGTCC-3′; CHOP, 5′- CCCTGCTTCTCTGGCTTGGCTGAC -3′ and 5′-GCTCCCAATTGTTCATGCTTGGTGC-3′; GAPDH, 5′-GACCCCTTCATTGACCTCAACTACATG-3′ and 5′-GTCCACCACCCTGTTGCTGTAGCC-3′; BiP, 5′-GAGTTCTTCAATGGCAAGGA-3′ and 5′-CCAGTCAGATCAAATGTACCC-3′; GRP94, 5′-CTGGGACTGGGAACTTATGAATG-3′ and 5′-TCCATATTCGTCAAACAGACCAC-3′. PCR products were resolved on 2% (w/v) agarose gels.

### In vitro transcription

cDNAs encoding human proteins were used for in vitro assays. The plasmids pCMV3-AFP, pMD-TF, pGEM-FGA, pCMV3-apoE and pGEM-ALB were obtained from Sino Biological. TF cDNA was subcloned into pcDNA3.1 using NheI and AgeI. An opsin tag (OPG2; NGTEGPNFYVPFSNKTG) containing two consensus N-glycan sites was inserted in-frame at the 3’-end of the indicated cDNAs by mutagenesis. pcDNA5-Sec61β_OPG2_, pBluescriptII-PDI and pBluescriptII-ERp57 were previously described^68,69^. pcDNA5-α1AT was provided by Cornelia Wilson (Canterbury Christ Church University, Kent, UK), pCMV6-GRP94 was a gift from Neil Bulleid (University of Glasgow, UK) and pcDNA3.1-BiP was bought from Addgene. All sequences were verified by sequencing (GATC, Eurofins Genomics). Linear DNA templates were generated by PCR using appropriate primers incorporating three additional Met codons at the end of the sequence to improve radiolabelling and transcribed into RNA with T7 polymerase (Promega).

### In vitro translocation assay

Translation was performed in rabbit reticulocyte lysate (Promega) supplemented with 1 mCi/ml EasyTag [^35^S]Met/Cys (PerkinElmer), amino acid mix minus Met (Promega), 0.1× volume of an in vitro transcribed mRNA (200–1000 ng/μl stock) and 6.5% (v/v) nuclease-treated ER microsomes (from stock with OD280 = 44/ml). Translation reactions were supplemented with Ipom-F 5% (v/v) from 20 μM stock solution in DMSO or an equal volume of DMSO and incubated for 30–60 min at 30 °C. AFP_OPG2_ and GRP94 were synthesised using the TNT Coupled Transcription/Translation system (Promega) for 90 min at 30 °C, as described by the manufacturer. Translation termination and ribosome release was induced with 1 mM puromycin for 10 min at 37 °C. Membrane-associated fractions were isolated by centrifugation through an 80 μl high-salt sucrose cushion (0.75 M sucrose, 0.5 M KOAc, 5 mM Mg(OAc)_2_, 50 mM Hepes–KOH, pH 7.9) at 100,000×*g* for 10 min at 4 °C in a TLA100 rotor (Beckman Coulter). Membrane pellets were resuspended directly in 2× SDS-PAGE sample buffer and, where indicated, treated with 500 units of EndoH (New England Biolabs) for 1 h at 37 °C prior to analysis. All samples were resolved by SDS-PAGE and radiolabelled products were visualised by phosphorimaging using Typhoon FLA-7000 (GE Healthcare). Band intensities were quantified using AIDA software (Raytest). Translocation was calculated by dividing the intensity of the N-glycosylated product by the intensity of the non-glycosylated product within each lane. This value was then expressed relative to the corresponding DMSO control in order to derive the relative ER translocation.

### Cytotoxicity assay

Cell viability was evaluated by using a 3-(4,5-dimethylthiazol-2-yl)-2,5-diphenyltetrazolium bromide (MTT) assay. Experiments were done in triplicate and were repeated three times. About 18,750 cells were seeded per well of a 96-well plate and incubated for 24 h. Ipom-F was dissolved in DMSO to make 1000× stock solutions. A series of working solutions of Ipom-F were prepared freshly by diluting the 1000× stock solutions in DMEM. Subsequently, the cells were treated with 200 μl of the freshly made working solution per well for 24 h. Cells were then washed twice with PBS, the media were discarded, and 200 μl of fresh complete medium containing 0.5 mg/ml MTT was added to each well. Following incubation at 37 °C for 3 h, media were discarded and 200 μl of DMSO was added to each well to dissolve formazan crystals. Absorbance of formazan was detected by a microplate reader (BioTek Synergy H1) at 570 nm with 650 nm as the reference wavelength. The percentage of viability compared to the negative control (DMSO-treated cells) was determined. Prism 8 (GraphPad) was used to make a plot of % viability versus sample concentration and to calculate the concentration at which a compound exhibited 50% cytotoxicity (IC50). IC50 value estimates were determined using non-linear regression to fit data to a curve of variable slope (four parameters).

### Statistical analysis

All experiments were repeated at least two times with representative data shown. Quantifications were analysed using Prism 8 (GraphPad). For multiple comparisons against a single control, statistical significance was determined using one-way ANOVA followed by Tukey’s comparison post hoc test against control.

## Supplementary Information

Supplementary Information.
