# Supplementary material for: Ipomoeassin-F disrupts multiple aspects of secretory protein biogenesis
Source: Sci Rep. 2021 Jun 2;11:11562. doi: 10.1038/s41598-021-91107-4 (PMC8173012; doi:10.1038/s41598-021-91107-4)
Supplement: Supplementary file 1 — Supplementary Information. [file 41598_2021_91107_MOESM1_ESM.pdf]

Supplementary Information

Ipomoeassin-F disrupts multiple aspects of secretory protein biogenesis

Peristera Roboti[1]*, Sarah O'Keefe[1], Kwabena B. Duah[2], Wei Q. Shi[2] and Stephen High[1]*

[1]Division of Molecular and Cellular Function, School of Biological Sciences, Faculty of Biology, Medicine and Health, The University of Manchester, Manchester M13 9PT, United Kingdom

[2]Department of Chemistry, Ball State University, Muncie, Indiana 47306, United States

*corresponding authors (peristera.roboti@manchester.ac.uk; stephen.high@manchester.ac.uk)

Supplementary Table 1: Primary antibodies used in this study

Antibody	Species	Source	Cat. No.	Application (dilution)
α-α1AT	rabbit	Dako	GA50561-2	WB (1/1000)<br>IF (1/100)
α-AFP	chicken	R&D Systems	AF1369	WB (1/1000)<br>IF (1/100)
α-TF	sheep	Philip Woodman, Manchester, UK		WB (1/1000)<br>IF (1/100)
α-FGA	mouse	Santa Cruz	sc-398806	WB (1/1000)
α-apoE	mouse	Santa Cruz	sc-13521	WB (1/1000)
α-ALB	rabbit	Sigma	A6684	WB (1/1000)
α-Stx5	rabbit	Synaptic Systems	110053	WB (1/5000)
α-TfR	mouse	Invitrogen	13-6800	WB (1/1000)
α-OST48	rabbit	In house		WB (1/1000)
α-Bag6	mouse	Abnova	H00007917-B01P	WB (1/1000)
α-BiP	goat	Santa Cruz	sc-1051	WB (1/1000)
α-GRP94	rat	Enzo	ADI-SPA-850	WB (1/1000)
α-Hsp70	mouse	Abcam	ab47455	WB (1/5000)
α-Hsc70	rat	Enzo	ADI-SPA-815	WB (1/1000)
α-Hsp90	rabbit	Enzo	ADI-SPA-846	WB (1/2000)
α-ATF4	rabbit	Cell Signaling	11815	WB (1/1000)
α-ATF6	rabbit	Cell Signaling	65880	WB (1/1000)
α-tubulin	mouse	Keith Gull, Oxford, UK		WB (1/1000)
α-TGN46	sheep	Vas Ponambalam, Leeds, UK		IF (1/400)
α-CRT	rabbit	David H. Llewellyn, Cardiff, UK		IF (1/100)

Supplementary Figure 1

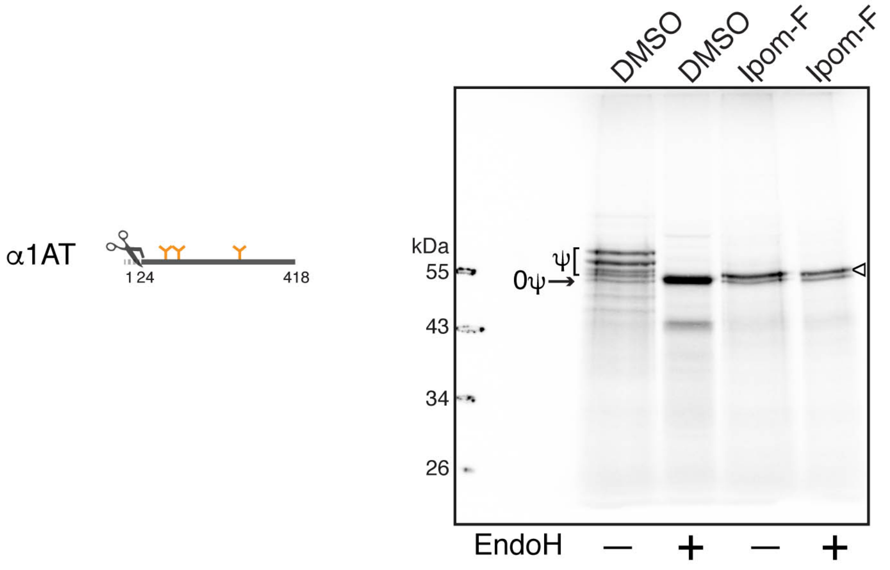

Supplementary Figure 1: Ipom-F reduces signal peptide-cleavage of α1AT *in vitro*. α1AT was translated in rabbit reticulocyte lysate supplemented with [^{35}S]Met/Cys, canine rough microsomes and either DMSO or Ipom-F (1 µM). Membrane-associated products were isolated by ultracentrifugation, resolved by SDS-PAGE and analysed directly by phosphorimaging. Samples were treated with EndoH to distinguish N-glycosylated (ψ) from non-glycosylated (0ψ) products. Ipom-F treatment produced non-translocated, signal peptide-containing precursor forms of α1AT (arrowhead) that were non-glycosylated, and therefore EndoH-resistant. Diagram of α1AT precursor is shown on the left. The cleavage site of its N-terminal signal peptide (scissors symbol) and its endogenous N-glycosylation sites (orange Y symbols) are indicated.

Supplementary Figure 2

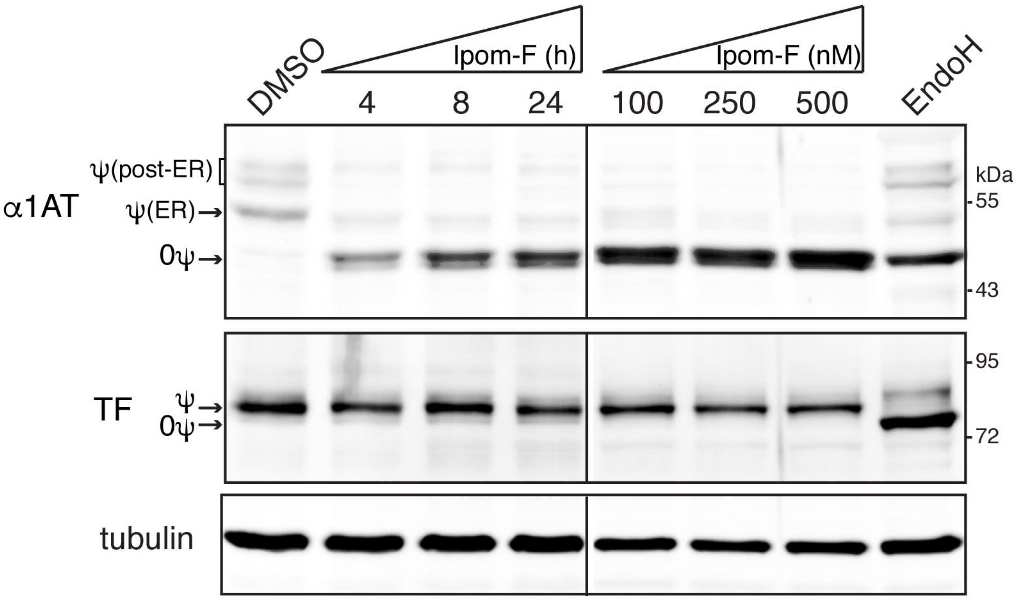

Supplementary Figure 2: Ipom-F does not impair N-glycosylation of TF under harsh treatment conditions. HepG2 cells were treated with DMSO, 100 nM Ipom-F over a time-course or with increasing concentrations of Ipom-F for 24 h. Clarified lysates were analysed by immunoblotting for the indicated proteins. EndoH digestion was performed to distinguish N-glycosylated (ψ) from non-glycosylated (0ψ) species. Post-ER, complex-glycosylated and ER resident, core-glycosylated α1AT products are shown. Solid line indicates different parts of the same gel. Full-length immunoblots are presented in Supplementary Figure 6.

Supplementary Figure 3

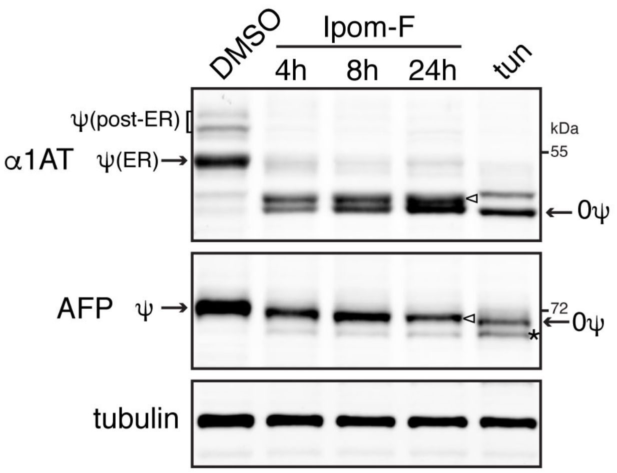

Supplementary Figure 3: The observed Ipom-F-mediated induction of stress-inducible cytosolic chaperones correlates with the appearance of non-imported secreted protein precursors. HepG2 cells were treated with DMSO for 24 h, tunicamycin (tun) for 17 h or Ipom-F for the indicated times. Detergent-soluble lysates were blotted for endogenous α1AT or AFP. Tunicamycin treatment effectively prevented N-glycosylation (indicated by ψ) to yield non-glycosylated (0ψ) species. Complex- and core-glycosylated α1AT products are shown. Non-glycosylated precursors of α1AT and AFP, whose ER import was inhibited by Ipom-F are indicated by an arrowhead. Note that these products migrate slightly slower than the corresponding ER-located precursors in tunicamycin-treated cells (0ψ), as predicted by signal peptide cleavage of the latter. The asterisk indicates a truncated form of AFP[1,2]. Full-length immunoblots are presented in Supplementary Figure 6.

Supplementary Figure 4

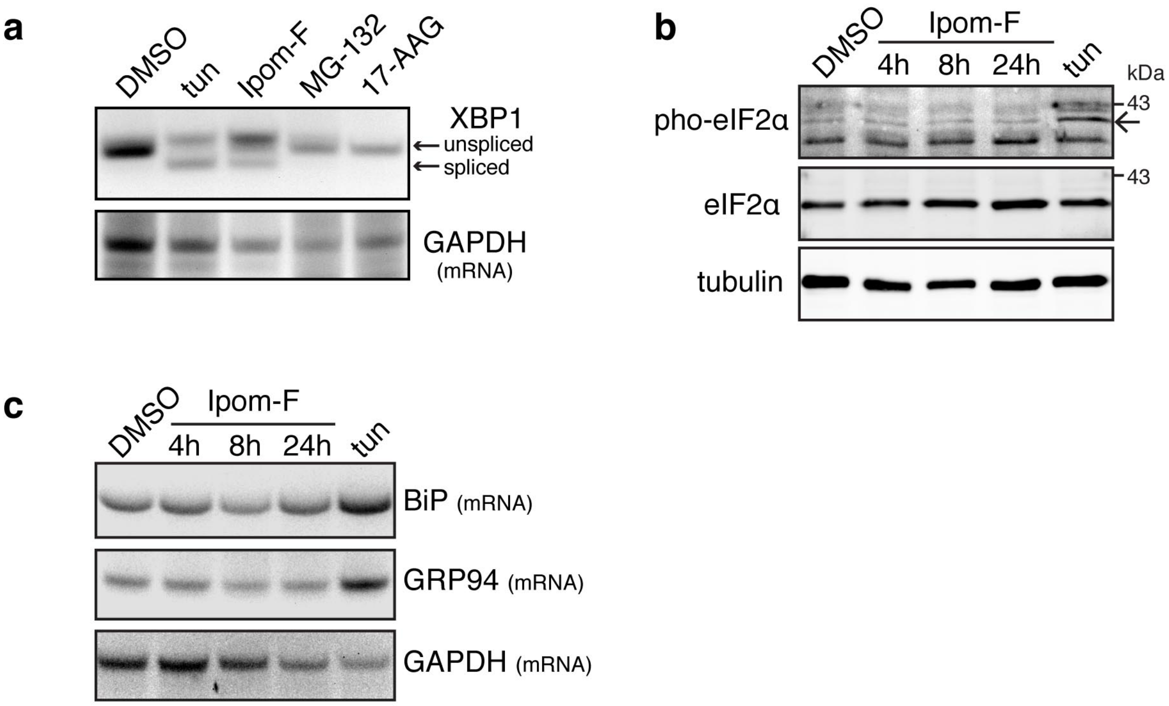

Supplementary Figure 4: Characterisation of Ipom-F-dependent ER stress.
(a) Cytosolic stress induced by the proteasome inhibitor MG-132 or the Hsp90-specific inhibitor 17-AAG does not activate the UPR. HepG2 cells were treated with DMSO, Ipom-F or 17-AAG for 24 h, with tunicamycin (tun) for 12 h or MG-132 for 4 h. XBP1 mRNA splicing was determined by reverse transcription-PCR. Unspliced and spliced XBP1 mRNA are indicated. GAPDH served as a cDNA loading control. **(b)** Phosphorylation of eIF2α cannot be detected in Ipom-F-treated cells. HepG2 cells were treated with DMSO for 24 h, tunicamycin (tun) for 12 h or Ipom-F for the indicated times. Detergent-soluble lysates were blotted with eIF2α phospho-specific antibodies using eIF2α and tubulin as loading controls. **(c)** ER stress triggered by Ipom-F-mediated Sec61 blockade does not cause transcriptional upregulation of BiP and GRP94. HepG2 cells were treated with DMSO for 24 h, tunicamycin (tun) for 12 h or Ipom-F for the indicated times. RNA was isolated from cells and mRNA levels of BiP and GRP94 was determined by reverse transcription-PCR. GAPDH served as a cDNA loading control. Full-length immunoblots and agarose gels are presented in Supplementary Figures 6 and 7, respectively.

Supplementary Figure 5

related to Figure 1a

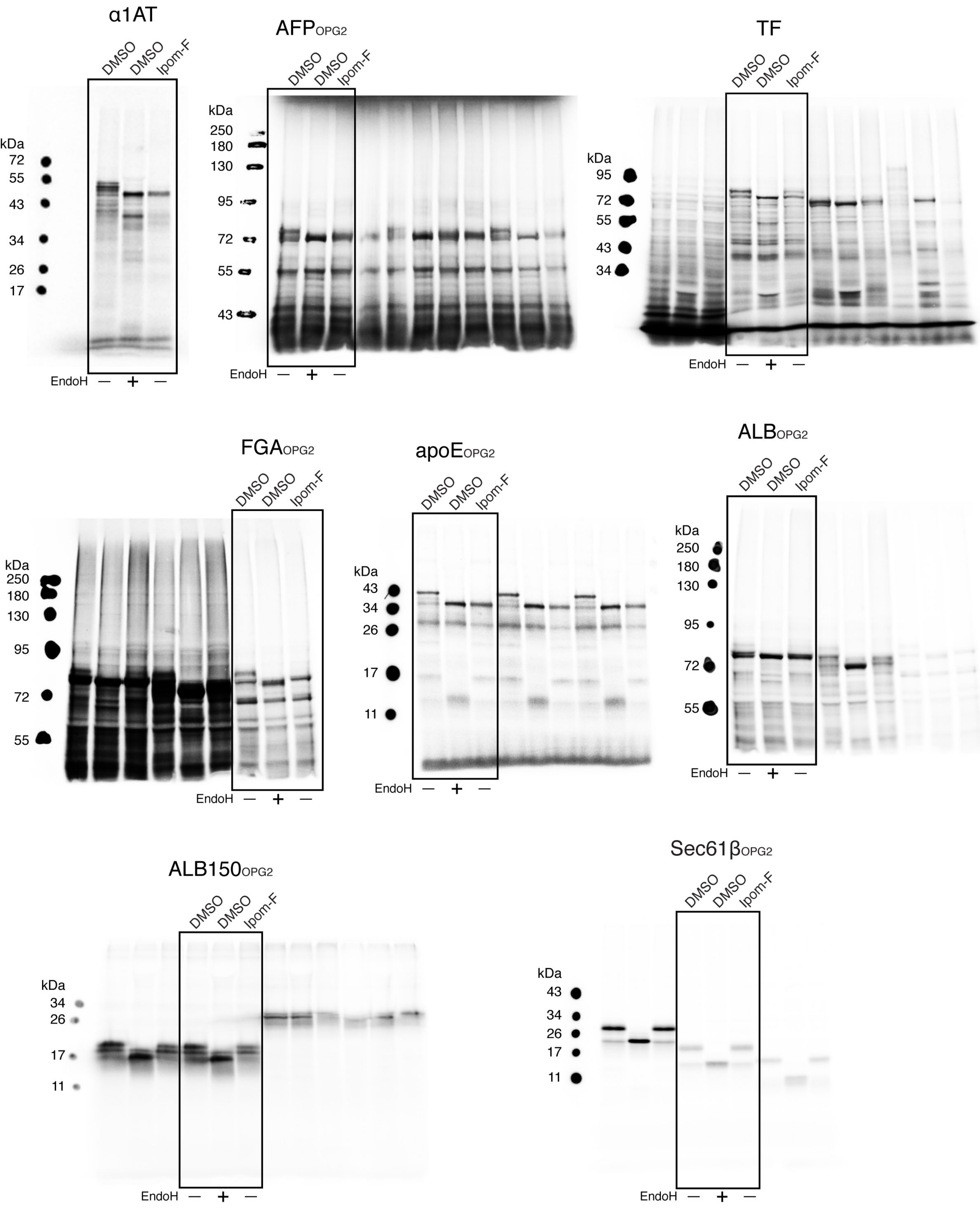

Supplementary Figure 5 (continued)

related to Figure 7a

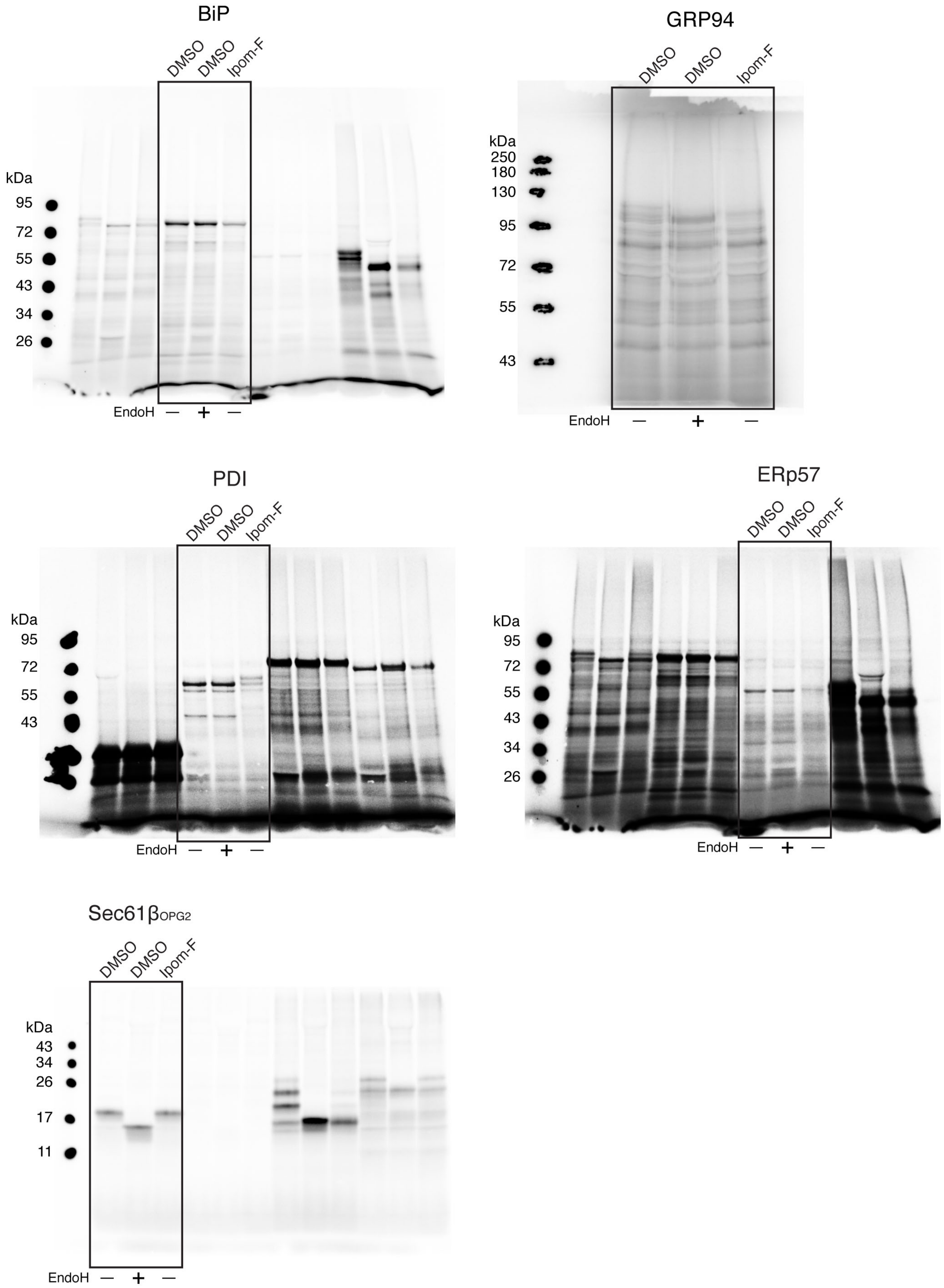

Supplementary Figure 5: Full-length phosphorimaging exposures from Figures 1a and 7a.

Supplementary Figure 6

related to Figure 2a

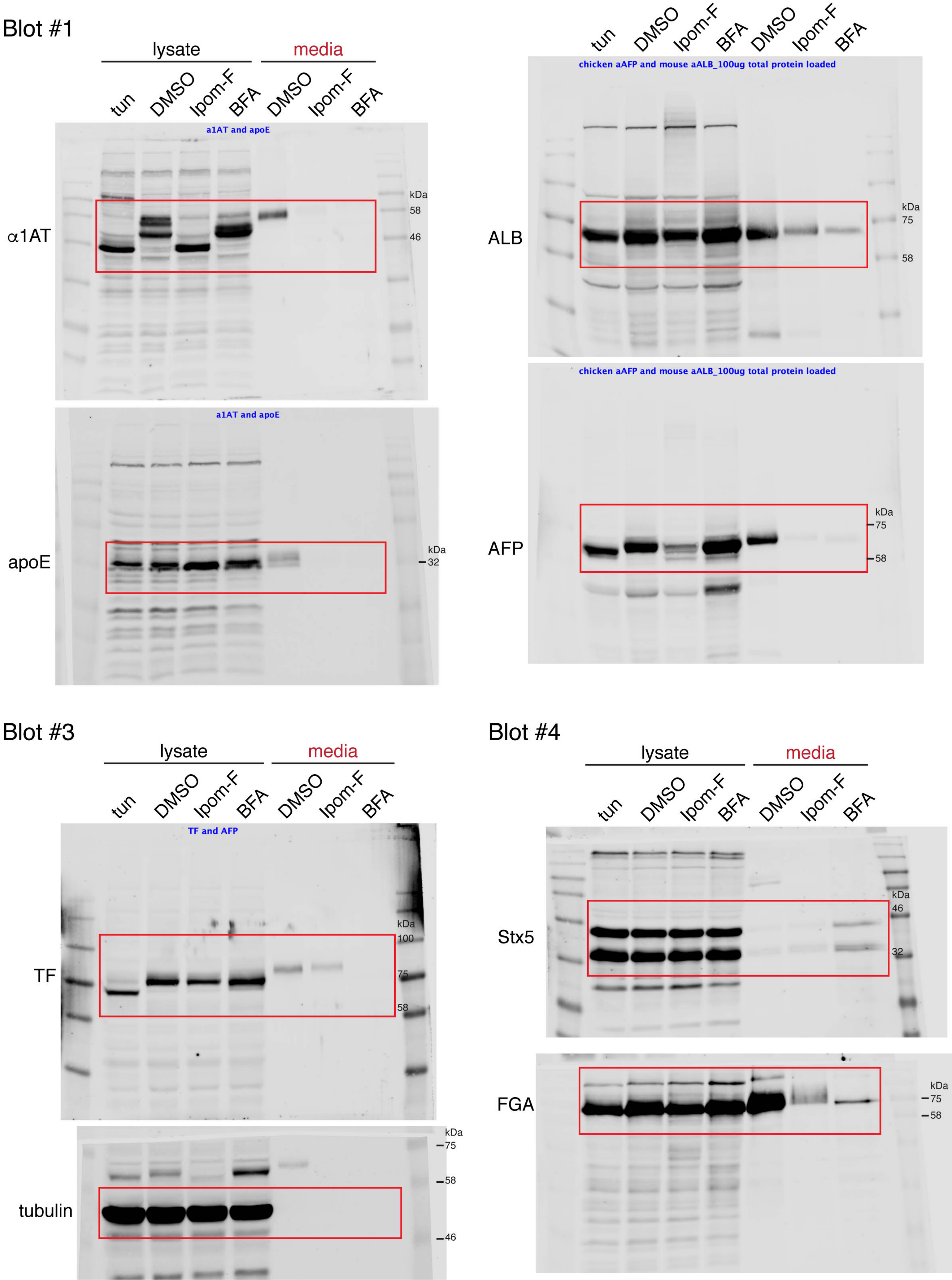

Supplementary Figure 6 (continued)

related to Figure 3a

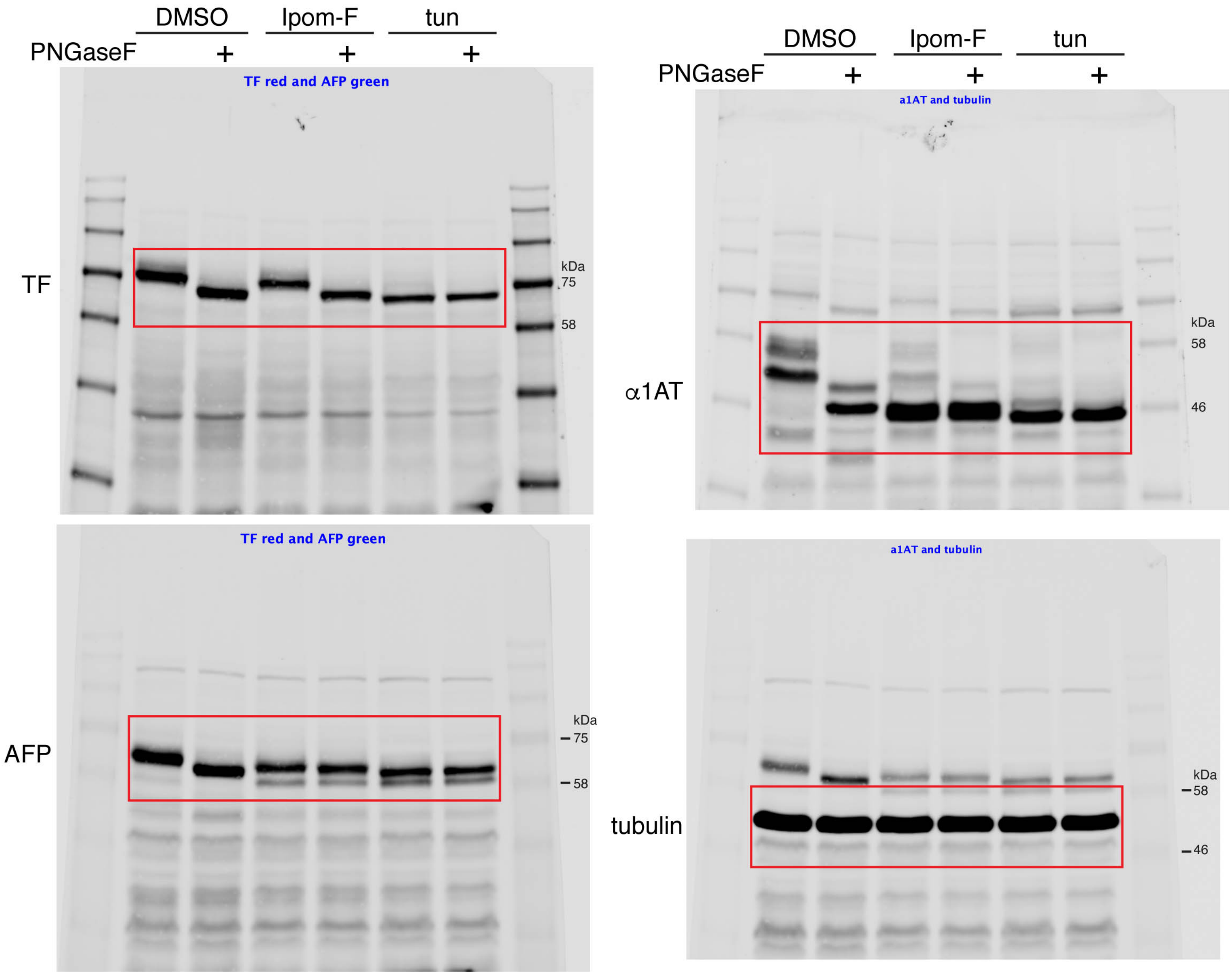

Supplementary Figure 6 (continued)

related to Figure 4a

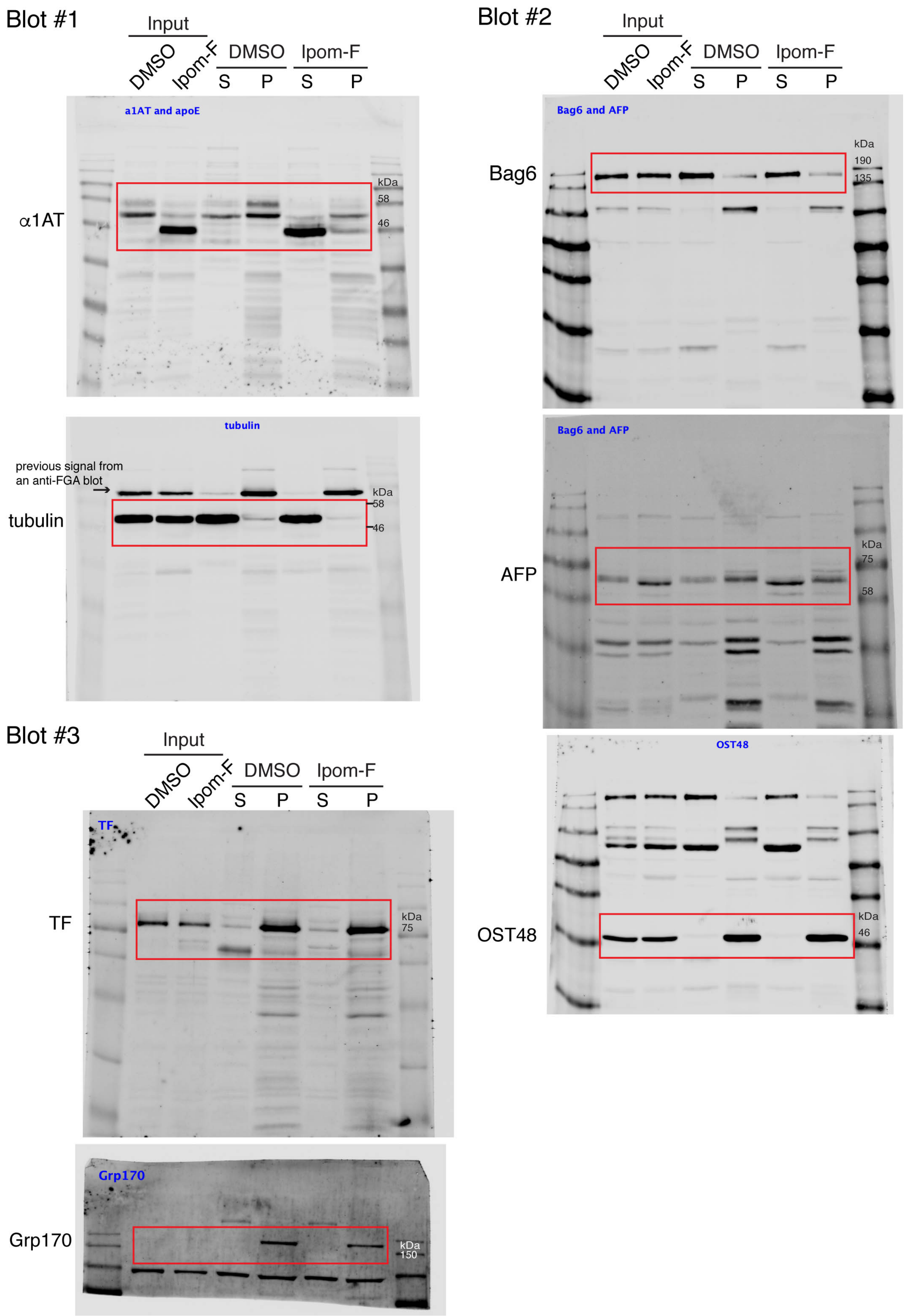

Supplementary Figure 6 (continued)

related to Figure 4c

Blot #1

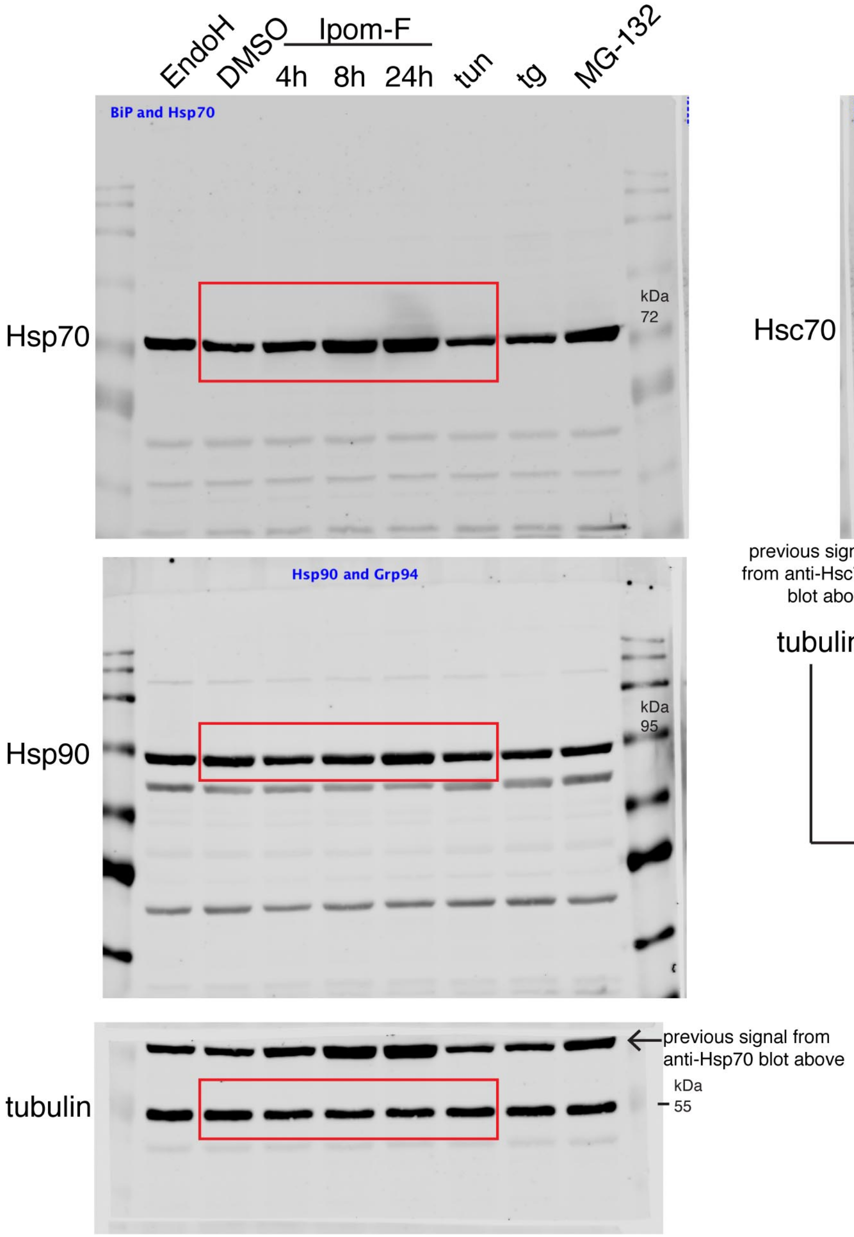

Blot #2

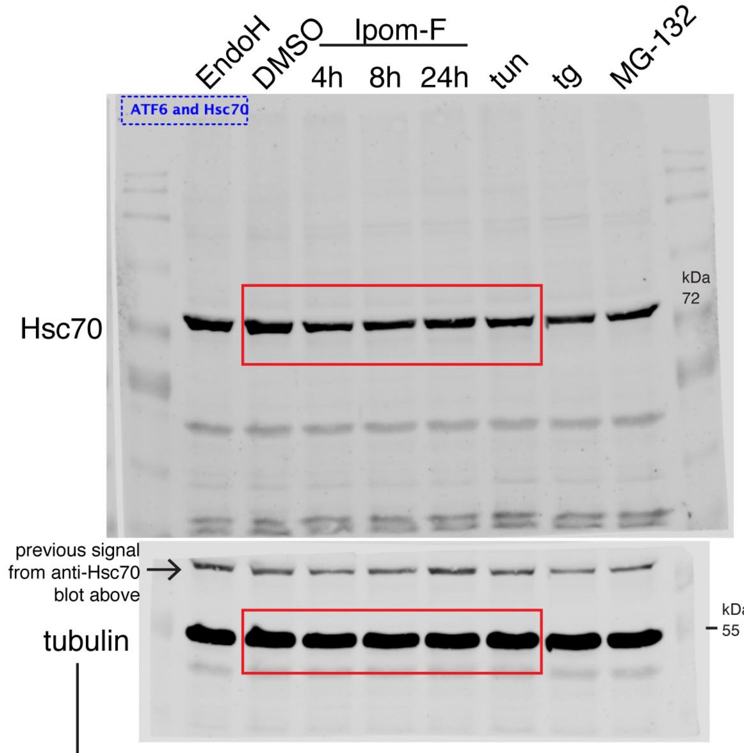

Please note that this tubulin loading control was also used for Figure 6e, where it accompanies the signal for ATF6.

Supplementary Figure 6 (continued)

related to Figure 5a

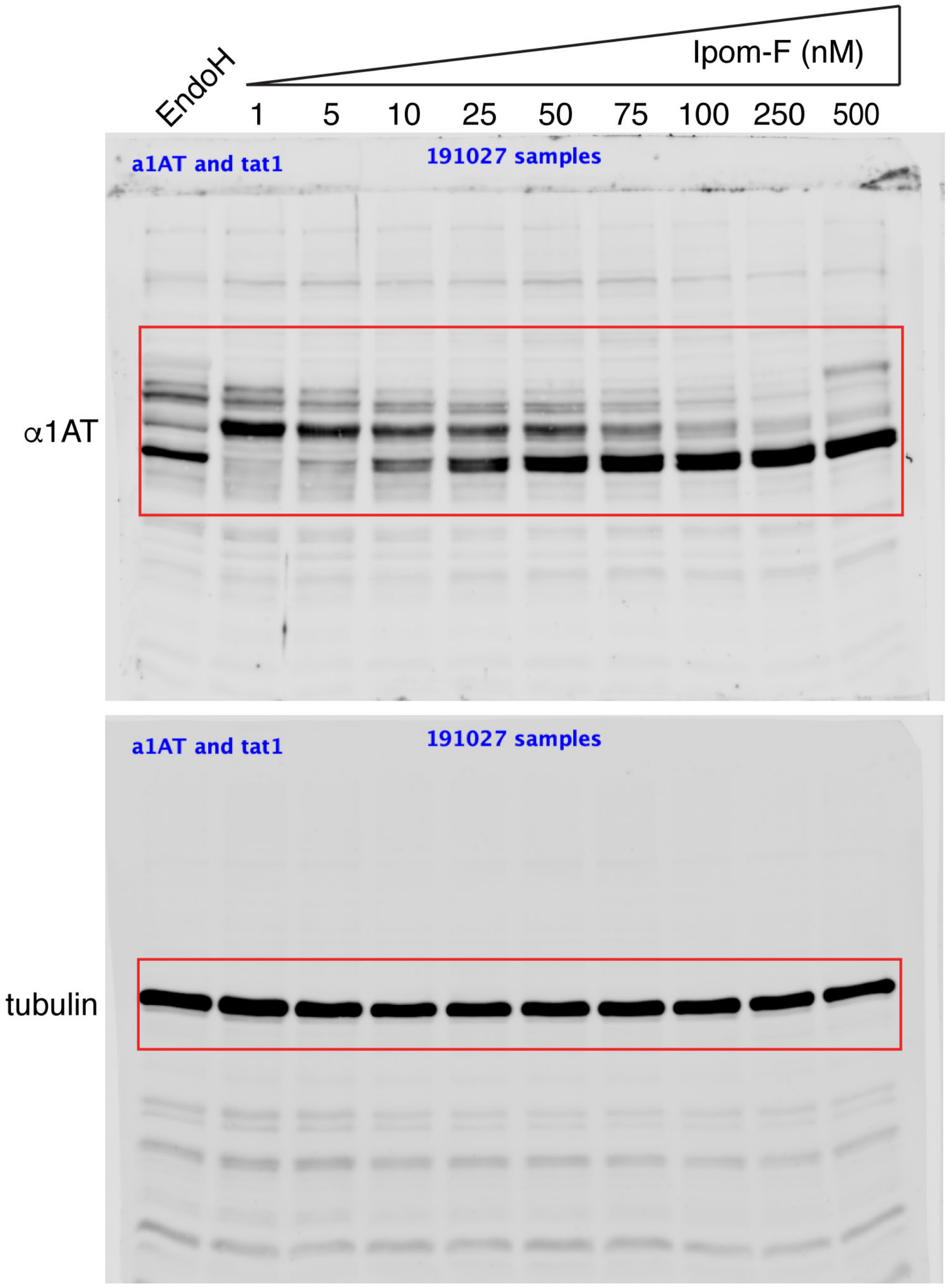

Supplementary Figure 6 (continued)

related to Figure 6b

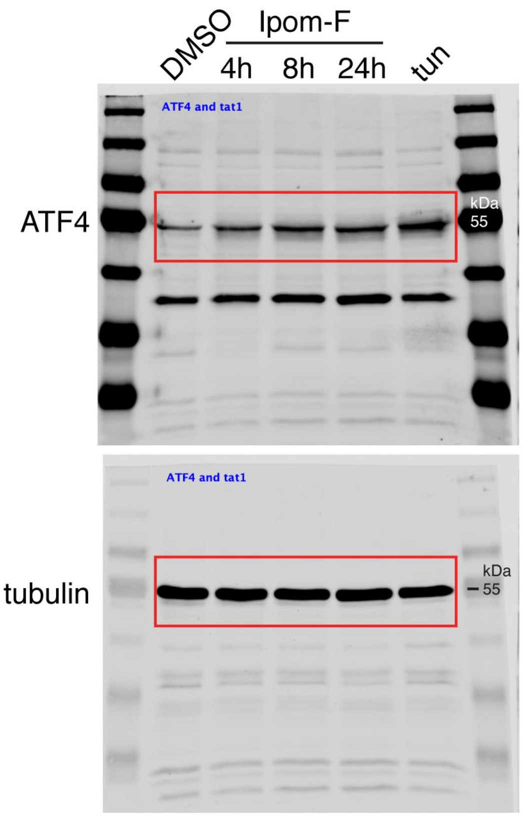

related to Figure 6c

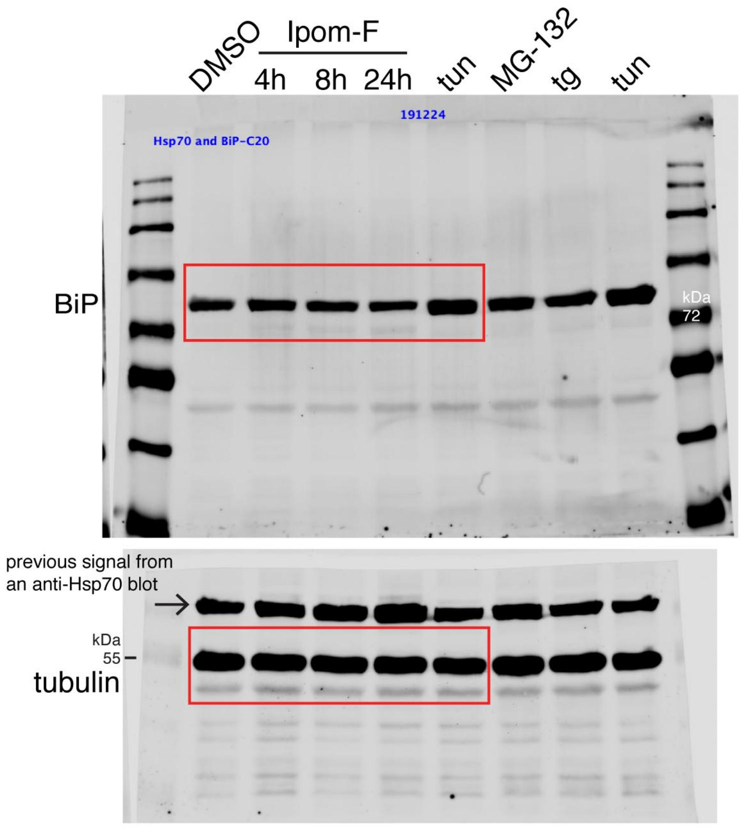

related to Figure 6d

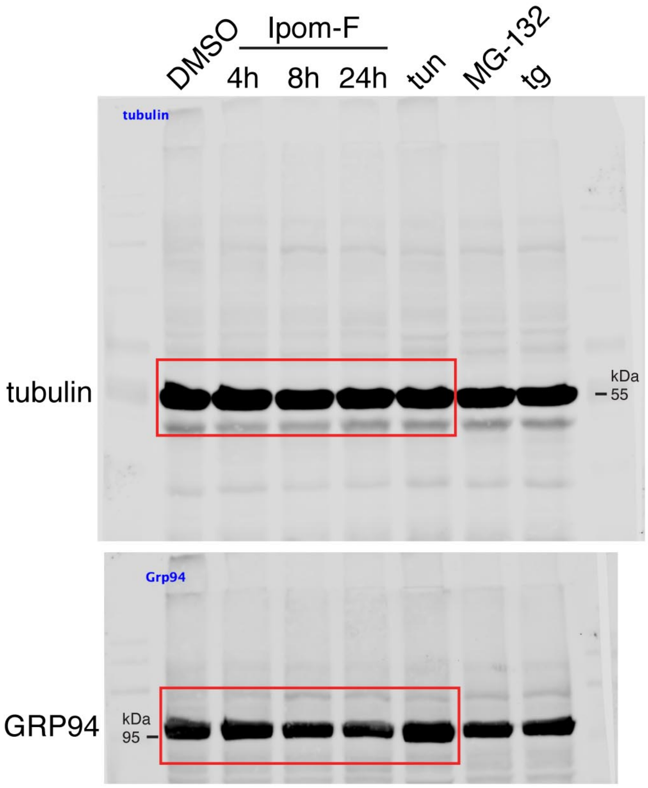

related to Figure 6e

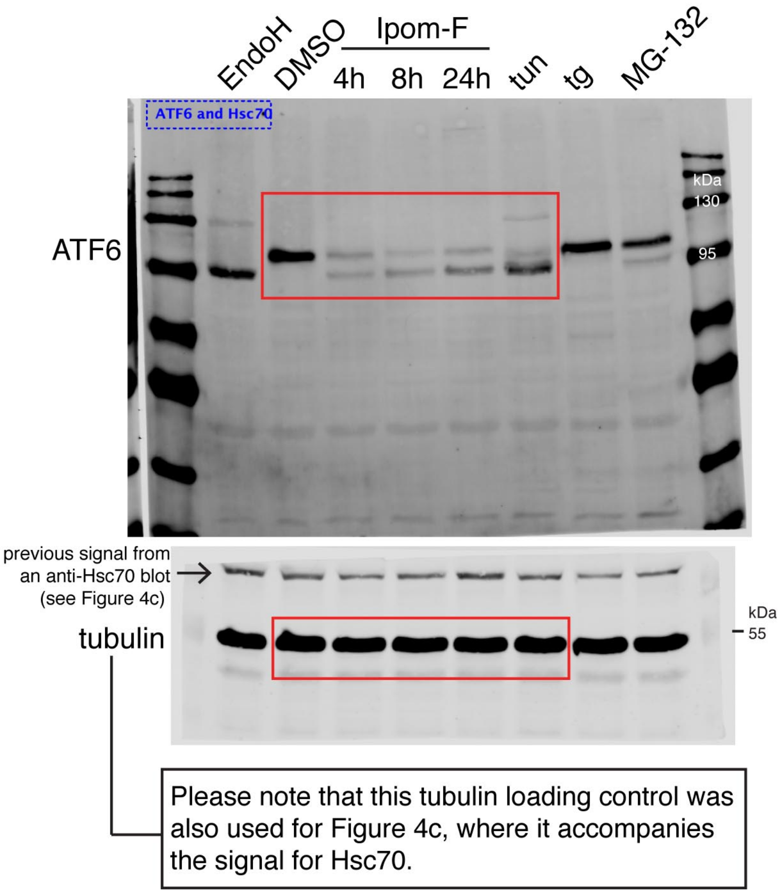

Please note that this tubulin loading control was also used for Figure 4c, where it accompanies the signal for Hsc70.

Supplementary Figure 6 (continued)

related to Supplementary Figure 2

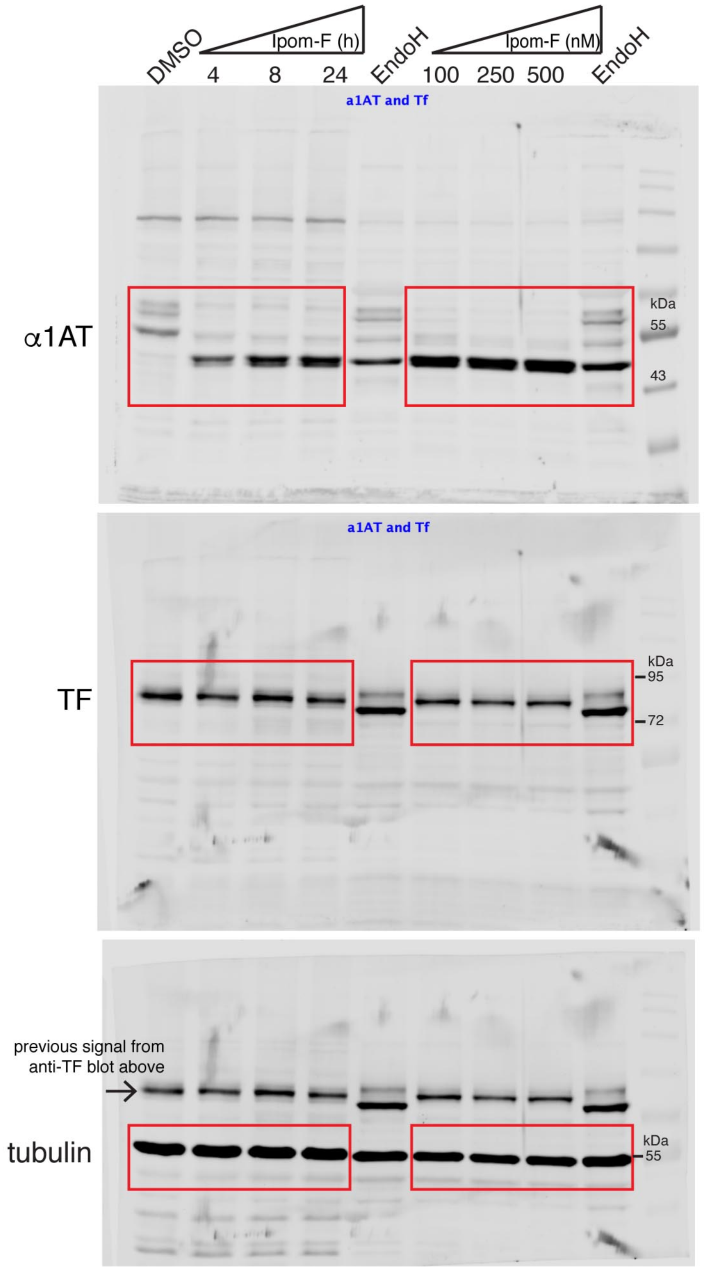

related to Supplementary Figure 3

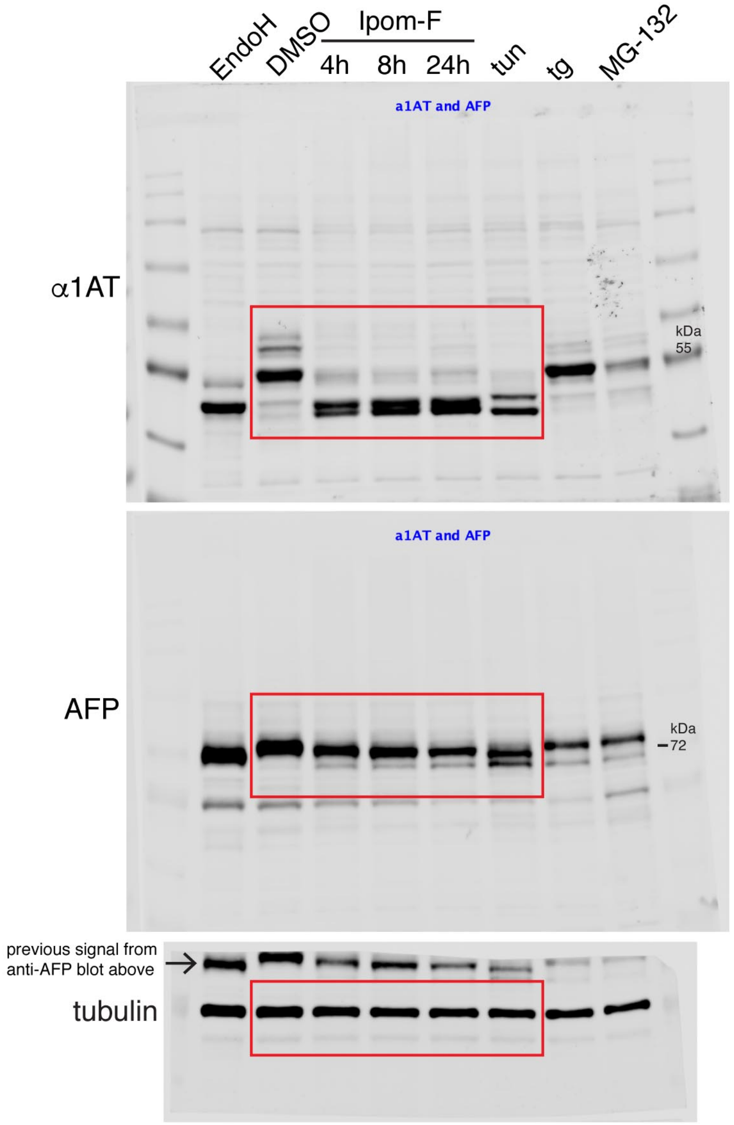

Supplementary Figure 6 (continued)

related to Supplementary Figure 4b

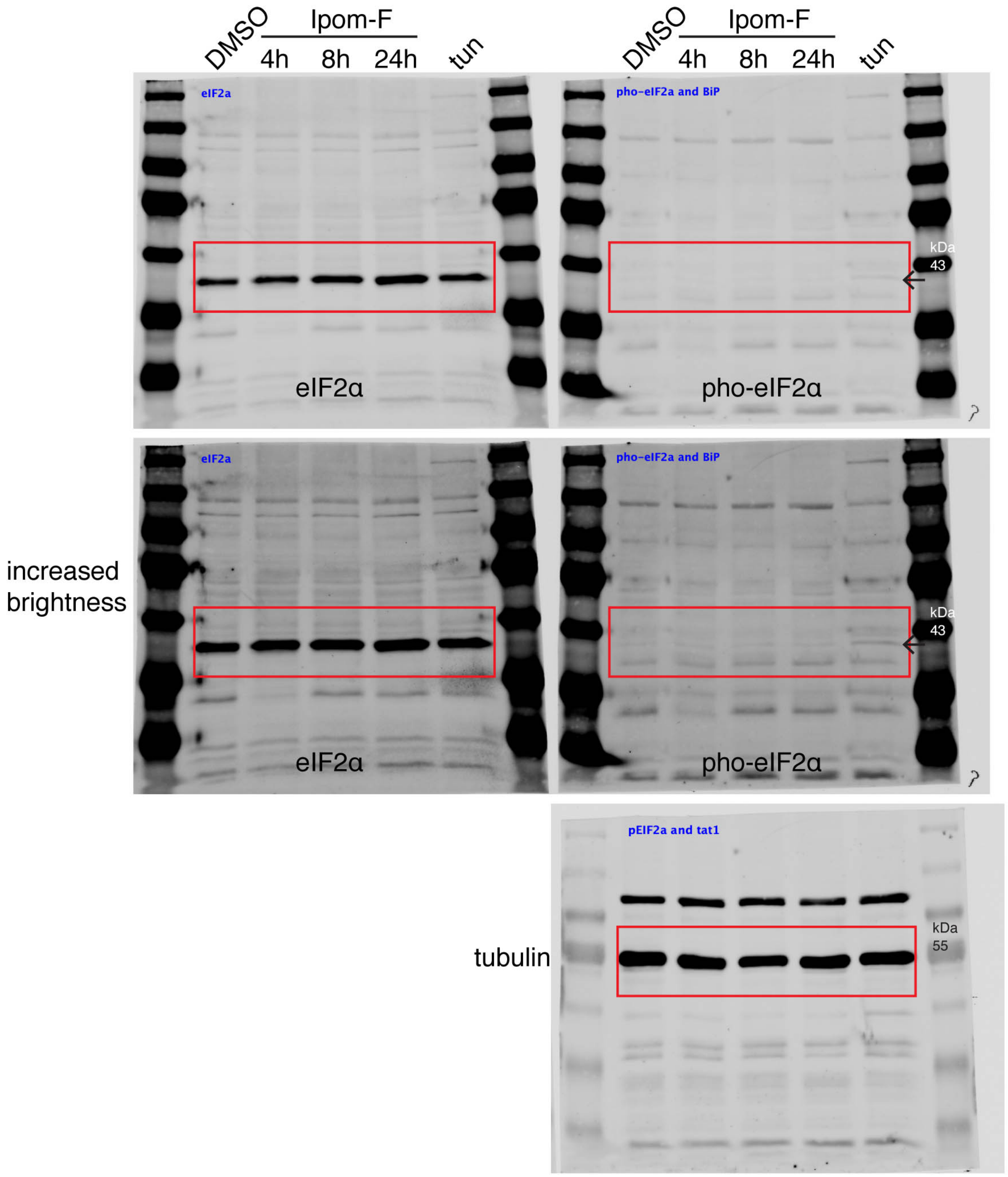

Supplementary Figure 6: Full-length, infrared-based scans of immunoblots from the indicated main and supplementary figures. The brightness of the entire scans was generally increased so that borders of membranes are easier to see. Please note that for Figures 4c and 6e, the same membrane was incubated with rat anti-Hsc70 and rabbit anti-ATF6 antibodies (two-colour detection system with Odyssey Infrared Imager), and therefore the same anti-tubulin immunoblot is shown in both figures.

Supplementary Figure 7

related to Figure 6a

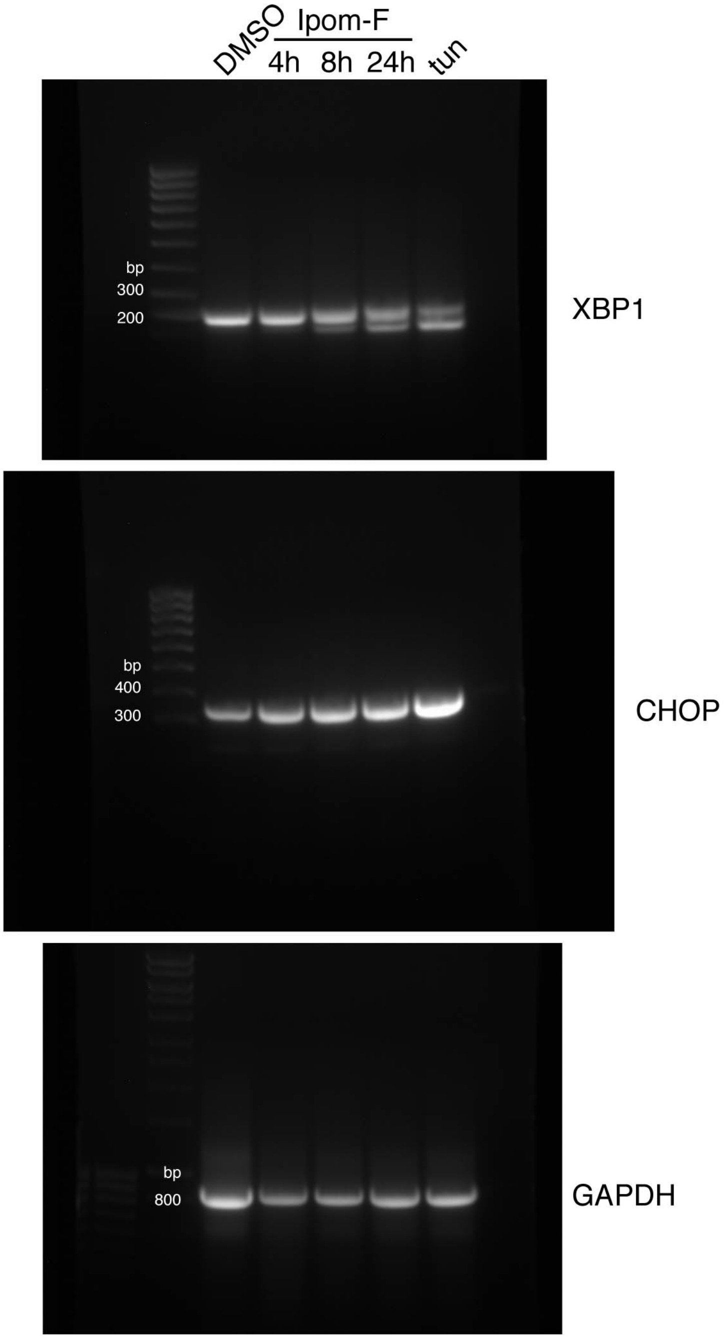

Supplementary Figure 7 (continued)

related to Supplementary Figure 4a

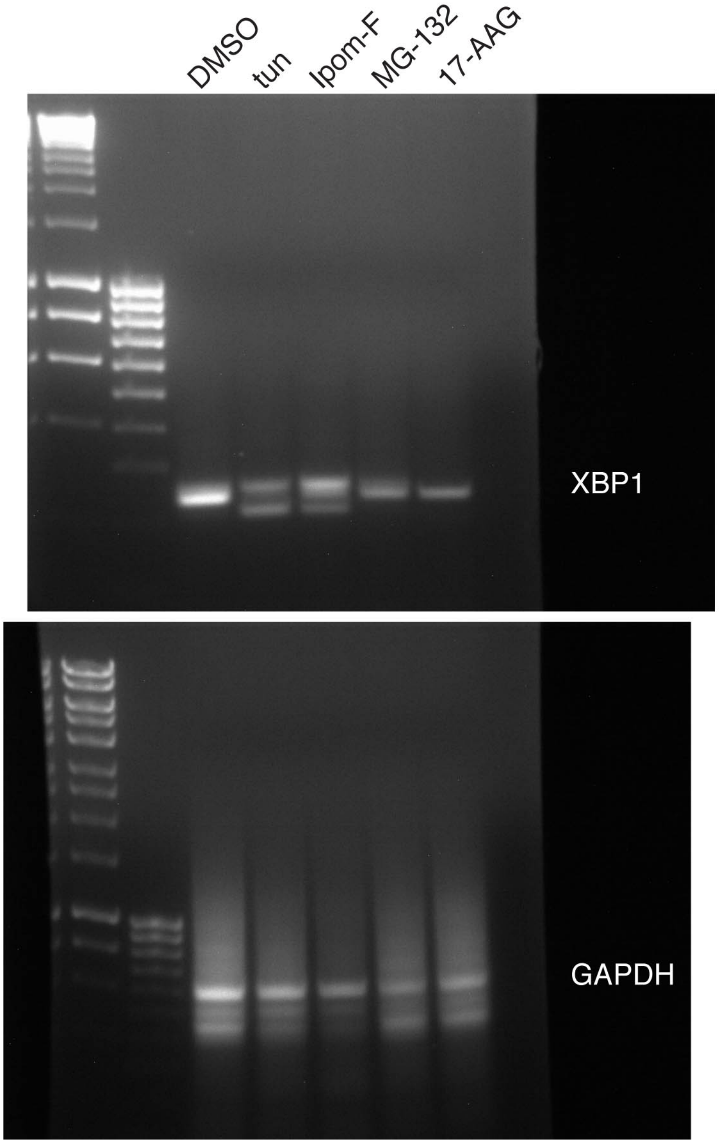

related to Supplementary Figure 4c

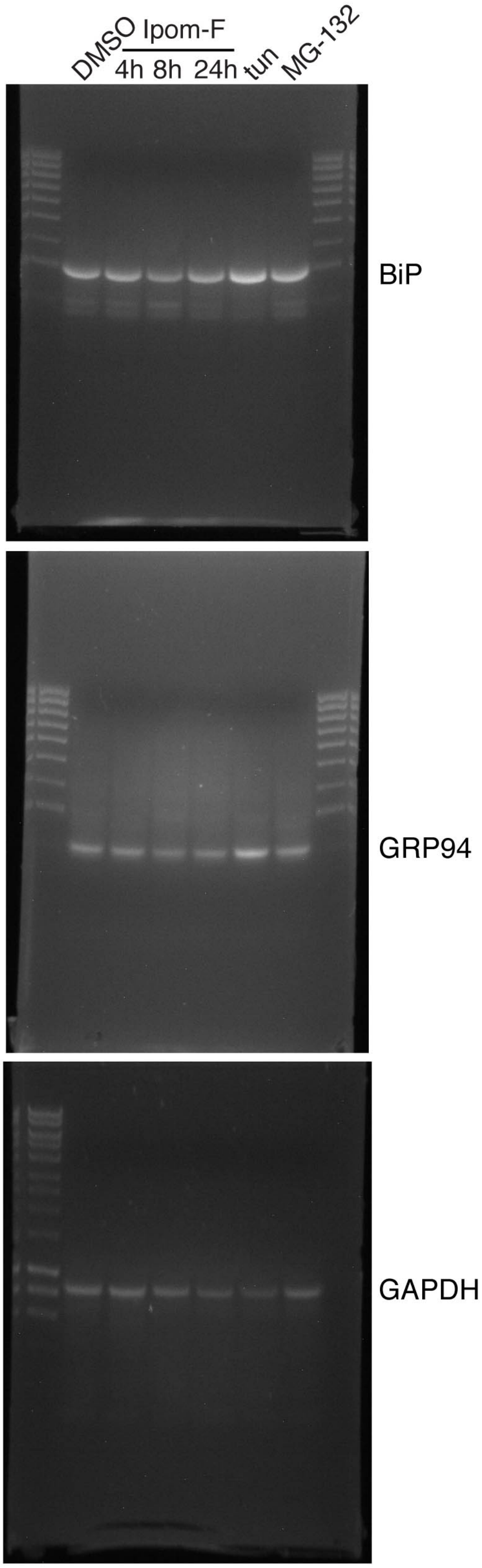

Supplementary Figure 7: Full-length scans of agarose gels from Figure 6a and Supplementary Figure 4a and c.

1 Deutsch, H. F., Taniguchi, N. & Evenson, M. A. Isolation and properties of human alpha-fetoprotein from HepG2 cell cultures. *Tumour Biol* **21**, 267-277, doi:10.1159/000030132 (2000).

2 Chou, J. Y. & Savitz, A. J. alpha-Fetoprotein synthesis in transformed fetal rat liver cells. *Biochem Biophys Res Commun* **135**, 844-851, doi:10.1016/0006-291x(86)91005-3 (1986).
